# Guidelines for the assessment and acceptance of potential brain-dead organ donors

**DOI:** 10.5935/0103-507X.20160049

**Published:** 2016

**Authors:** Glauco Adrieno Westphal, Valter Duro Garcia, Rafael Lisboa de Souza, Cristiano Augusto Franke, Kalinca Daberkow Vieira, Viviane Renata Zaclikevis Birckholz, Miriam Cristine Machado, Eliana Régia Barbosa de Almeida, Fernando Osni Machado, Luiz Antônio da Costa Sardinha, Raquel Wanzuita, Carlos Eduardo Soares Silvado, Gerson Costa, Vera Braatz, Milton Caldeira Filho, Rodrigo Furtado, Luana Alves Tannous, André Gustavo Neves de Albuquerque, Edson Abdala, Anderson Ricardo Roman Gonçalves, Lúcio Filgueiras Pacheco-Moreira, Fernando Suparregui Dias, Rogério Fernandes, Frederico Di Giovanni, Frederico Bruzzi de Carvalho, Alfredo Fiorelli, Cassiano Teixeira, Cristiano Feijó, Spencer Marcantonio Camargo, Neymar Elias de Oliveira, André Ibrahim David, Rafael Augusto Dantas Prinz, Laura Brasil Herranz, Joel de Andrade, Associação de Medicina Intensiva Brasileira, Associação Brasileira de Transplante de Órgãos

## Abstract

Organ transplantation is the only alternative for many patients with terminal diseases. The increasing disproportion between the high demand for organ transplants and the low rate of transplants actually performed is worrisome. Some of the causes of this disproportion are errors in the identification of potential organ donors and in the determination of contraindications by the attending staff. Therefore, the aim of the present document is to provide guidelines for intensive care multi-professional staffs for the recognition, assessment and acceptance of potential organ donors.

## Abstract (pt)

O transplante de órgãos é a única alternativa para muitos pacientes portadores de algumas doenças terminais. Ao mesmo tempo, é preocupante a crescente desproporção entre a alta demanda por transplantes de órgãos e o baixo índice de transplantes efetivados. Dentre as diferentes causas que alimentam essa desproporção, estão os equívocos na identificação do potencial doador de órgãos e as contraindicações mal atribuídas pela equipe assistente. Assim, o presente documento pretende fornecer subsídios à equipe multiprofissional da terapia intensiva para o reconhecimento, a avaliação e a validação do potencial doador de órgãos.

## INTRODUCTION

Organ transplantation is often the only therapeutic option for patients with end-stage failure of different vital organs. However, there is a worrisome disproportion between the demand for organ transplants and the number of transplants that are actually performed in Brazil and other countries.

The Brazilian medical authorities are actively seeking to minimize the discrepancy between the supply and demand for organs. Many of the problems on the supply side derive from flaws in the recognition of brain death, interaction with potential donor relatives, clinical maintenance of brain-dead donors and determination of contraindications. Although the corrective measures seem obvious, this problem does not receive due attention in most intensive care units (ICU) in Brazil, as evidenced by the almost absolute absence of systematics for the care of potential multiple-organ donors. This matter is beyond the mere technical sphere; it is a humanitarian and civic issue that concerns all actors involved in the maintenance of brain-dead potential donors, among whom intensivists should play a leadership role. The lack of robust evidence on the subject strongly points to the relevance of formal orientations (even when merely consensus-based in many respects) to have a minimum uniformity in the performance of protocols for the assessment and acceptance of brain-dead potential donors. Given the weakness of the available evidence, it is important to note that divergences in the recommendations formulated by the *Conselho Federal de Medicina* (CFM) are indeed a possibility. In any case, the CFM recommendations should be followed.

The present guidelines discuss essential aspects of the protocol for the assessment and acceptance of brain-dead potential donors and seek to provide grounds for the diagnosis of brain death and determination of the eligibility of potential multiple-organ donors.

The aim of the present guidelines is to contribute to intensive care and institutional transplant coordination through uniform care standards for brain-dead donors to optimize organ transplantation both quantitatively and quantitatively based on measures that are applicable to Brazilian society.

## METHODS

Preliminary questions were formulated based on an extensive literature review conducted by an ad hoc Writing and Planning Committee composed of doctors from the *Associação de Medicina Intensiva Brasileira* (AMIB) and the *Associação Brasileira de Transplante de Órgãos* (ABTO). The preliminary questions were sent to all authors as a starting point for suggestions, replacements and the formulation of new questions. The questions were revised by the Executive Committee and then sent to the authors for text development.

The primary sources consulted were located in the following databases: Scientific Electronic Library Online (SciELO), Embase and MEDLINE^®^, using the PubMed search engine. The search was performed to answer questions styled according to the PICO (Population, Intervention, Comparison, Outcome) method. The following search terms selected from the MeSH (Medical Subject Headings) database were used: (potential brain-death donor OR brain death diagnosis OR brain death definitions OR brain death criteria AND consensus), (potential brain-death donor OR brain death diagnosis AND clinical test OR ancillary test), (organ donor OR potential brain-death donor AND mechanical ventilation OR strategies of ventilation OR apnea test), (potential brain-death donor OR brain death diagnosis AND lazarus phenomenon OR lazarus sign), (potential brain-death donor OR brain death diagnosis AND sedation OR sedatives OR drugs OR neuromuscular blockers OR anesthetics OR neurologic depressors), (potential brain-death donor OR expanded criteria donors), (potential brain-death donor OR clinical evaluation AND expanded criteria donors OR expanding the donor pool), (potential brain-death donor OR organ donation AND increased infectious risk donors OR infectious disease OR virus transmisson OR bacterial transmission OR fungal transmission), (potential brain-death donor OR organ donation AND tumoral disease OR cancer AND neoplasia), (potential brain-death donor OR organ donation AND older adults OR aging), (organ donor OR lung transplantation AND contraindications AND expanded criteria donors), (organ donor OR kidney transplantation AND contraindications AND expanded criteria donors), (organ donor OR liver transplantation AND contraindications AND expanded criteria donors OR expanding the donor pool), (brain-death OR organ donor AND renal donation), (renal function AND brain-death organ donation), (brain-death organ donor AND kidney transplantation), (organ transplantation OR donor kidneys OR management donor kidneys), (transplantability AND liver OR hepatic AND donor), (cadaveric donor AND timing AND liver transplantation), (expanding the donor pool AND liver OR marginal donor liver AND outcome OR extended criteria donor AND MELD), (deceased cardiac donor AND cardiac transplantation AND contraindications AND expanded criteria donors OR expanding the donor pool).

Given the nature of the present document, laws, bills, decrees, ordinances and resolutions were also considered bibliographical references. The located references were subjected to critical analysis and categorized according to strength of the evidence and the recommendations formulated by the Guidelines Project of the *Associação Médica Brasileira* (AMB) and CFM (Table 1).

**Table 1:** Method for grading the quality of evidence and defining the strength of the recommendations

Quality of scientific evidence per study type*	
A	Experimental or observational studies with greater consistency
	1A: Systematic reviews (with homogeneity) of randomized controlled trials
	1B: Individual randomized controlled trials with narrow confidence intervals
	1C: “All or none” randomized controlled trials
B	Experimental or observational studies with lower consistency
	2A: Systematic reviews (with homogeneity) of cohort studies
	2B: Individual cohort study (including low quality randomized controlled trials)
	2C: Outcomes research; ecological studies
	3A: Systematic reviews (with homogeneity) of case-control studies
	3B: Individual case-control studies
C	Case series (and poor quality cohort and case-control studies)
D	Expert opinion without explicit critical appraisal, or based on physiology, bench research or “first principles”
Strength of the recommendations according to GRADE**	
Strong	Must be followed (we recommend)
Weak	Perhaps should be followed (we suggest)
Unspecific	There are neither advantages nor disadvantages

Sources:

* Associação Médica Brasileira - AMB, Conselho Federal de Medicina - CFM. Projeto Diretrizes. São Paulo: AMB/CFM; 2001. Available at http://www.projetodiretrizes.org.br/projeto_diretrizes/texto_introdutorio.pdf;

** Guyatt GH, Oxman AD, Vist GE, Kunz R, Falck-Ytter Y, Alonso-Coello P, Schünemann HJ; GRADE Working Group. GRADE: an emerging consensus on rating quality of evidence and strength of recommendations. BMJ. 2008;336(7650):924-6. GRADE: Grading of Recommendation, Assessment, Development and Evaluation.

Topics were divided into four subgroups: (1) concepts and screening for potential donors, (2) diagnosis of brain death, (3) criteria for potential donor selection and (4) organ-specific contraindications.

Texts written based on the formulated questions were organized by the Writing and Planning Committee, reviewed by the Executive Committee and returned to the authors for revision. Once the final text was produced, it was distributed to all participants and discussed at two plenary meetings held in May and October 2015.

The project chairs presented recommendations, which were then discussed. As the strength of the evidence underlying a large part of the recommendations was low, the grading criteria of the Grading of Recommendations Assessment, Development and Evaluation (GRADE) (Table 1) were adopted. GRADE categorizes recommendations as strong (should be followed), weak (perhaps should be followed) or unspecific (no advantages or disadvantages). In a strong recommendation for a given intervention, the desired effects clearly outweigh the undesirable effects. In a weak recommendation for a given intervention, the desired effects are likely to outweigh the undesirable effects, but the group making the recommendation is not completely confident, either because some of the evidence is low quality or because additional studies are needed. In the case of an unspecific recommendation, its benefits and disadvantages are balanced and thus must be evaluated on a case-by-case basis. Strong recommendations should be understood as "we recommend" and weak recommendations as "we suggest".

## PART 1: CONCEPTS AND SCREENING FOR POTENTIAL DONORS

### 1. How to define brain death?

**Comment:** Brain death is defined as the irreversible loss of all functions of the brain, including the brainstem (**D**).^(1-6)^ Brain death is equivalent to death, despite the maintenance of a heartbeat and spinal cord function (**D**).^(1)^ The diagnosis of brain death demands proof of irreversible loss of consciousness, brainstem reflexes and the ability to breath (**D**).^(5)^

**Recommendation:** Brain death is defined as the irreversible loss of brain functions (including the brainstem) manifested by unresponsive coma, absence of brainstem reflexes and apnea (**D**). **Strong Recommendation**.

### 2. What criteria and terminology define a patient as likely to become an organ and tissue donor?

**Comment:** The most common causes of brain death are traumatic brain injury (TBI) and stroke, which account for more than 90% of potential organ donors. Other causes include brain tumors, central nervous system (CNS) infections and post cardiac arrest anoxic brain injury (**B**).^(7,8)^ Historically, TBI was the main cause of brain death in Brazil; however, this situation is changing in some of the country's states.

Before progressing to brain death, many patients exhibit a state known as "imminent brain death" from which they might pass to the status of possible organ donors (**D**).^(9)^ This condition should be clearly defined and recognized in critically ill patient care services, and it should be emphasized that these patients are not brain dead and thus must receive the required intensive care until irreversibility is confirmed.

In 2008, a group of specialists from the World Health Organization (WHO) and The Transplantation Society (TTS) harmonized the terminology for the donation-transplantation process. After three meetings, the new glossary was presented as a WHO recommendation in 2010 (**D**).^(10)^ This harmonization was necessary because terms were used in many different ways, thus hindering the comparison of outcomes between countries.

The terminology recommended by WHO and TTS is as follows:

- Possible donor: patient with a devastating brain injury or lesion sustained with mechanical ventilation (**D**).^(10)^
- Potential donor: a person whose clinical condition is suspected to fulfill brain death criteria, i.e., a patient is considered a potential donor the moment the brain death protocol is started.
- Eligible donor: the diagnosis of brain death is confirmed, and there are no previously known contraindications to donation.
- Actual donor: a person in whom an operative incision was made with the intent of organ recovery.
- Utilized donor: an actual donor from whom at least one organ was transplanted.

**Recommendation:** Individuals with severe brain injury or brain death must be classified according to the terminology formulated by the WHO (**D**). **Weak Recommendation**.

### 3. Should there be a strategy for the systematic search for possible donors or brain-dead individuals? What clinical criteria are considered essential for such a systematic search? What protocol features are considered essential? Who should apply the protocol?

**Comment:** The Third WHO Global Consultation on Organ Donation and Transplantation called on each country to strive to achieve self-sufficiency in organ transplants **(D)**.^(11)^ The resulting document also instructed countries to maximize donation through the application of adequate protocols for end-of-life care. The WHO call agrees with the principle asserting that decision-making on end-of-life care should be based on an assessment of the patient's best interests, which go beyond his or her physical needs to encompass broader issues such as social, ethical and moral aspects, including the desire to donate organs. Successful donation programs essentially depend on the identification and notification of all potential donors. Equally important is the recognition of the occurrence of brain death as soon as possible to institute effective maintenance measures and reduce the risk of family refusal **(D)**.^(11,12)^

Until 2010, more than half of the individuals who died in a state of brain death in Brazil were not identified, considering an underestimated rate of 70 brain death cases per 1 million inhabitants per year. Since then, slightly more than 70% of cases have been identified, i.e., three out of 10 cases of brain death in Brazil are not identified **(C)**.^(13)^ Similar results have been reported in Europe and Canada **(C)**^(14)^ **(D)**.^(15)^ The ACCORD Consortium (Achieving Comprehensive Coordination in Organ Donation throughout the European Union) found that 35% of the patients who died by devastating brain injury at European hospitals were not reported as such, and thus the possibility of organ donation could not even be considered **(D)**.^(14)^

Therefore, a systematic search of individuals with brain death is crucial to correct identification flaws **(D)**.^(16,17)^ The process of identification of possible and potential donors ideally should include the following features:

1. Determination of sites where possible donors are usually found. All hospital sectors with patients under invasive mechanical ventilation, especially critically ill patient care services.
2. Accurate knowledge of the defined criteria for possible and potential donors. Identification and notification of the In-hospital *Comissão Intra-hospitalar de Doação de Órgãos e Tecidos para Transplante* (CIHDOTT) and the *Central de Captação, Notificação e Distribuição de Órgãos e Tecidos do Estado* (CNCDO) of all possible donors fulfilling the following criteria: under mechanical ventilation, with devastating irreversible brain injury of known origin, score of 3 on the Glasgow Coma Scale and absence of one or more brainstem reflexes (D);^(12,18)^ and all individuals who fulfill the brain death criteria formulated by the CFM and described in question 4.
3. Establishment of the minimum frequency of the active search, which is twice per day **(D)**.^(16,17)^
4. Identification of ICU team leaders and transplant coordinators presenting the conditions required to systematize the identification of brain-dead individuals. In institutions with established transplant coordination, an active search must be performed by transplant coordinators (doctors or nurses). In all other institutions, an active search should be conducted by professionals with broad experience in the management of neurocritical care patients **(D)**.^(16,17)^ Active participation of intensive care providers in the donation process is essential because it sensitizes the staff to organ transplantation, promotes education and training and facilitates interactions between ICU staff and in-hospital transplant coordination.

**Recommendation:** Daily rounds should focus on the identification of brain-dead individuals and possible donors with devastating irreversible brain injury, score of 3 on the Glasgow Coma Scale and absence of one or more brainstem reflexes **(D)**. **Strong Recommendation**.

**Recommendation:** Daily rounds conducted to identify possible donors should be performed systematically by transplant coordinators and/or the professionals in charge of units that provide care to critically ill patients, in all sectors with mechanical ventilators **(D)**. **Strong Recommendation**.

**Recommendation:** If there are no clinical contraindications, discontinue sedation for some time every day and assess possible donors with devastating irreversible brain injury during this period **(D)**. **Weak Recommendation**.

**Recommendation:** As a suggestion, rounds should be performed at least twice per day **(D)**. **Weak Recommendation**.

**Recommendation:** Notify all potential donors to CIHDOTT, *Organização de Procura de Órgãos* (OPO) or your state's CNCDO **(D)**. **Strong Recommendation**.

**Recommendation:** Notify all possible donors with devastating irreversible brain injury, score of 3 on the Glasgow Coma Scale and absence of one or more brainstem reflexes to CIHDOTT, OPO or your state's CNCDO **(D)**. **Weak Recommendation**.

## PART 2: DIAGNOSIS OF BRAIN DEATH

### 4. What are the clinical criteria for the diagnosis of brain death?

**Comment:** To comply with law no. 9,434, the CFM passed resolution no. 1,480, which "establishes criteria to characterize Brain Death" **(D)**.^(18,19)^ This resolution defines criteria, procedures and steps for the determination of brain death. On legal and ethical grounds, the diagnosis of brain death should rigorously comply with the stipulations in both legal documents.

Article 4 of CFM resolution no. 1,480 establishes that "The clinical parameters required for determination of brain death are: unresponsive coma with absence of supraspinal motor activity, absence of brainstem reflexes and apnea". The appendix titled "Declaration of Brain Death" describes the "elements on neurological examination" that - in the absence of irreversible causes of coma - are confirmatory of brain death, which is incompatible with life.

Clinical confirmation of brain death requires the following:

1. The presence of unresponsive coma due to a well-defined cause, in the absence of spontaneous movements and of supraspinal motor responses to stimuli applied to the area of distribution of the cranial nerves on both sides of the body **(D)**.^(2,18-20)^

**Important:** Some brain-dead individuals might exhibit spinal reflexes, which do not suffice to exclude the diagnosis of brain death **(D)**.^(18,20)^ (See question 10).

2. Absence of brainstem reflexes **(D)**:^(2,18-20)^
  *Pupillary reflex:* absence of contraction of the iris sphincter muscle in response to light on both eyes, resulting in medium-sized or dilated fixed pupils.
  *Corneal reflex:* absence of response to stimulation of the cornea by touching.
  *Oculocephalic reflex:* absence of eye movements upon rotating the head to the sides.
  *Vestibulo-ocular response:* absence of eye movements on the caloric test.
  *Cough reflex:* absence of the cough reflex.
3. Absence of respiratory efforts confirmed by the apnea test **(D)**.^(2,18,19,21)^

**Recommendation:** Clinical diagnosis of brain death requires the presence of unresponsive coma of known cause, absence of all brainstem reflexes (pupillary, corneal, oculocephalic, vestibulo-ocular and cough reflex) and apnea **(D)**. **Strong Recommendation**.

### 5. How to clinically assess coma in a patient with suspected brain death?

**Comment:** Prior to the assessment of coma in the strict sense, some prerequisites should be fulfilled to rule out coma by reversible causes (see question 10): (1) presence of irreversible brain injury of known etiology able to cause the condition; (2) absence of evidence of exogenous intoxication or use of CNS depressants; (3) absence of severe hydroelectrolytic or acid-base abnormalities that are not due to the condition that caused coma but that might be the cause of coma; (4) core temperature ideally ≥ 35ºC (core blood, rectal, bladder or esophageal temperature); and (5) mean arterial pressure (MAP) ≥ 60mmHg or systolic arterial pressure (SAP) ≥ 100mmHg. Brain-dead individuals must exhibit unresponsive coma, with an absence of supraspinal activity, an absence of brainstem reflexes and apnea **(D)**.^(2,3,6,22)^

Coma must be assessed based on the presence or absence of motor responses to standardized painful stimuli, such as supraorbital and/or temporomandibular joint and/or nail bed pressure. The individual should not exhibit evidence of supraspinal motor responses to painful stimuli, but spinal reflexes might be present.

**Recommendation:** Rule out reversible causes of coma and verify the absence of supraspinal motor responses to standardized painful stimuli (supraorbital and/or temporomandibular joint and/or nail bed pressure) **(D)**. **Strong Recommendation**.

### 6. How should the brainstem reflexes be tested?

**Comment:** The brainstem reflexes should be assessed as follows **(D)**:^(2,3,6,20,22)^

*Pupillary reflex:* documented absence of response to light in both eyes, usually with fixed medium-sized or dilated pupils. Preexisting pupil abnormalities or prior surgery might interfere with the assessment.

*Corneal reflex:* absence of response to stimulation of the cornea by touching it with a non-traumatic device (e.g., 1 drop of 0.9% saline and cotton).

*Oculocephalic reflex:* presence of cervical spine injury must be ruled out first. While holding the patient's eyes open, the head is briskly turned from side to side; when the reflex is absent, the eyes rotate to the same side as the head and do not move within the orbits.

*Vestibulo-ocular reflex:* first confirm that the tympanum is intact and the external auditory meatus is clear. Performance of this test is not recommended in the presence of signs of basilar skull fracture. The patient's head is kept in a neutral position and elevated to 30º; 50mL of ice water (caloric test) are irrigated into the external auditory meatus; when the reflex is absent, the eyes do not move after a 1-minute observation. Each ear should be separately tested with a 5-minute interval. Smaller water volumes should be used for children under 2 years of age.

*Cough reflex:* absence of cough during gentle stimulation of the tracheal carina by inserting an aspiration cannula through the orotracheal tube.

**Recommendation:** Once the presence of unresponsive coma is established, all reflexes involving the cranial nerves should be tested (pupillary, corneal, oculocephalic, vestibulo-ocular and cough reflex), and the presence of apnea should be assessed according to a standardized technique **(D)**. **Strong Recommendation**.

### 7. How should the apnea test be performed?

**Comment:** To perform the apnea test, the patient must have normal blood pressure, normal body fluid content and temperature, satisfactory oxygenation and a partial pressure of carbon dioxide (PaCO_2_) of 40 - 45mmHg. The test assesses the absence of ventilatory drive in the presence of carbon dioxide (CO_2_) retention. The minimum PaCO_2_ level should be ≥ 60mmHg according to American guidelines and ≥ 50mmHg according to British recommendations, while the Canadian guidelines recommend a PaCO_2_ ≥ 60mmHg and ≥ 20mmHg increase over the baseline level **(D)**.^(6,22,23)^ The post-test PaCO_2_ level recommended in Brazil is ≥ 55mmHg **(D)**.^(19)^

The test should be performed as follows **(D)**:^(3,6,23,24)^

- Keep SAP ≥ 100mmHg.
- Pre-oxygenate by ventilation with a fraction of inspired oxygen (FiO_2_) of 100% for 10 minutes.
- Adjust the ventilator frequency to attain normocapnia (40 - 45mmHg).
- Collect an arterial blood sample for arterial blood gas testing.
- Disconnect the ventilator.
- Introduce a catheter through the tracheal tube up to the carina and administer oxygen at a flow rate of 6L/minute.
- Observe the patient for respiratory movements for 8 to 10 minutes.
- Stop the test if SAP < 90mmHg, oxygen saturation (SatO_2_) < 85% or cardiac arrhythmia develops.
- Collect a new blood sample for arterial blood gas testing.
- Reconnect the ventilator, reduce FiO_2_ (sufficient to maintain SaO_2_ > 90%) and reset the ventilation parameters to the pretest levels. Alveolar recruitment maneuvers might be needed after performance of the apnea test.^(25)^

Alternative options for patients who cannot tolerate being disconnected from the ventilator include the following:

1. Connect a T-piece coupled to a continuous positive airway pressure (CPAP) valve to the orotracheal tube and ventilate at a CPAP of 10cmH_2_O and an oxygen flow rate of 12L/minute **(B)**.^(26)^
2. Perform the apnea test using noninvasive ventilation equipment that permits a supplemental oxygen flow; set CPAP to 10cmH_2_O and the oxygen flow rate to 10 - 12L/minute. The apnea test should not be performed when the ventilator cannot provide the desired oxygen flow rate when operating in CPAP mode because it will cause hypoxemia **(D)**.^(25,27,28)^

If respiratory efforts are absent and the post-test PaCO_2_ is ≥ 55mmHg, then the test result is compatible with brain death **(D)**.^(19)^

The apnea test results should be interpreted cautiously in the case of patients with severe lung disease who were CO_2_ retainers (previous hypercapnia) **(D)**.^(23)^ A PaCO_2_ of 55 - 60mmHg may not suffice as a respiratory stimulus when the baseline PaCO_2_ is slightly lower. In such a situation, consider variations greater than 20mmHg over the baseline PaCO_2_ in addition to PaCO_2_ ≥ 55mmHg **(D)**.^(26)^

**Recommendation:** The apnea test must not last longer than 10 minutes and should be monitored by a doctor at the bedside. The test is considered to be positive for brain death when spontaneous respiratory efforts are absent with PaCO_2_ ≥ 55mmHg **(D)**. **Strong Recommendation**.

**Recommendation:** When the test is stopped before 10 minutes have elapsed, the results should be interpreted as follows:

- PaCO_2_ ≥ 55mmHg: compatible with brain death **(D).Strong Recommendation.**
- PaCO_2_ < 55mmHg: inconclusive (test must be repeated) **(D). Strong Recommendation**.

**Recommendation:** Set the ventilatory parameters to attain a PaCO_2_ of 40 - 45mmHg. For patients with severe lung disease, higher baseline PaCO**_2_**levels are acceptable **(D)**. **Weak Recommendation**.

**Recommendation:** The apnea test can be performed using three different techniques.

1. The patient is disconnected from the ventilator and administered oxygen at 6 L/minute **(D)**. **Weak Recommendation**.
2. The patient is disconnected from the ventilator and administered oxygen at 6L/minute with CPAP at 10cmH_2_O **(B)**. **Weak Recommendation**.
3. Using a specific noninvasive ventilation device, CPAP is set to 10cmH_2_O, and the oxygen flow rate is set to 10 - 12L/minute **(D)**. **Weak Recommendation**.

### 8. Who should perform the clinical tests for brain death and what is the minimum interval between clinical tests?

**Comment:** Article 3 of law no. 9,434 (February 4, 1997), also known as the Transplant Bill, stipulates that "Postmortem removal of tissues, organs or parts of a human body for transplantation or treatment should be preceded by a diagnosis of brain death established and recorded by two doctors not belonging to the removal and transplantation teams, based on clinical and technological criteria described in resolutions of the *Conselho Federal de Medicina*" **(D)**.^(19,29)^

According to decree no. 2,268, from June 30, 1997, a diagnosis of brain death should be confirmed by at least two doctors, one of whom is a specialist in neurology **(D)**.^(29)^ The order of examination is irrelevant, i.e., it does not matter whether the neurologist performs the first or the second examination. A later CFM ruling established that a diagnosis of brain death may be performed by a neurosurgeon or a pediatric neurologist instead of a specialist in neurology.

The minimum interval between the two clinical assessments required for the determination of brain death varies according to the age of the individual as follows **(D)**:^(19)^ 48 hours for infants aged 7 days to under 2 months old; 24 hours for infants aged 2 months to under 1 year old; 12 hours for infants aged 1 to under 2 years old; and 6 hours for individuals over 2 years old.

The interval between clinical assessments varies considerably at the global level **(D)**.^(30,31)^ Although CFM resolution no. 1,480 regulates the duration of this interval, the time interval defined for the diagnosis of brain death per age range is arbitrary **(D)**.^(3)^

**Recommendation:** Until laws establishing new criteria for the determination of brain death are passed, the clinical tests for the confirmation of brain death should be performed by at least two different doctors, one of whom should be a neurologist or neurosurgeon, at intervals established according to the age range **(D)**. **Strong Recommendation**.

### 9. Do spinal reflexes exclude a diagnosis of brain death? What is their frequency? Can neuromuscular blockers be used to inhibit spinal reflexes?

**Comment:** The essential criteria for the determination of brain death are complete unresponsiveness, an absence of brainstem reflexes and permanent apnea **(D)**.^(2)^ However, a variety of reflex movements was observed in brain-dead individuals, such as plantar flexion and extension responses, muscle stretch reflexes, abdominal reflexes and finger jerks **(D)**.^(32)^ Because these are spinal reflexes, their presence does not exclude a diagnosis of brain death.

According to some reports, reflex movements occur in more than 75% of brain-dead individuals. The frequency and type of these movements vary according to the triggering stimuli and the cause of the underlying brain injury. Reflex upper limb pronation and extension, abdominal contractions, finger jerks, periodic leg movements and the Lazarus sign (a complex spinal reflex that occurs in brain dead individuals characterized by arm and at times also trunk flexion during the apnea test or passive head movement) have been reported **(B)**.^(33)^

While several hypotheses have been proposed to account for the occurrence of these reflex movements, the underlying mechanisms are not yet fully understood. According to one hypothesis, the reflex movements represent hypoxia- and hypercapnia-induced activity of cervical cord neurons **(D)**.^(32)^ Alternatively, they might be due to disinhibition of movement generators of the spinal cord. Another hypothesis is that mechanical compression/decompression of the spinal root or cervical spinal cord by neck flexion/extension can generate movement **(B)**.^(33)^ The same intact spinal cord that exhibits spinal reflexes to noxious stimuli is also capable of inducing the release of catecholamines through the adrenergic loop, with deleterious systemic consequences when the uncontrolled sympathetic outflow is not prevented or treated **(D)**.^(34)^

Use of neuromuscular blocking agents can be considered in the pre- and transoperative management of brain-dead organ donors to prevent spinal reflexes in response to perioperative stimulation and once a definitive diagnosis of brain death is established **(D)**.^(34)^

**Recommendation:** Do not exclude a diagnosis of brain death due to the occurrence of spinal reflexes **(B)**. **Strong recommendation**.

**Recommendation:** Spinal reflexes can be inhibited through the use of muscle relaxants once the diagnosis of brain death is established **(D)**. **Weak Recommendation**.

### 10. Which reversible causes of unresponsive coma should be excluded?

**Comment:** The first step to excluding reversible causes of unresponsive coma is to objectively determine the etiology of coma based on the patient's medical history, physical examination, neuroimaging and laboratory tests. Once the etiology of coma is established, five conditions that are frequently mentioned in the literature as brain death mimics must be proactively investigated and excluded, namely (1) use of CNS depressants; (2) severe metabolic disorders; (3) severe hypothermia; (4) severe hypotension; and (5) drugs or diseases that cause motor paralysis.

Use of CNS depressants is discussed in full detail in question 11. Severe metabolic disorders, demonstrated by laboratory values that deviate markedly from the normal range and include blood glucose, electrolytes (sodium, phosphorus and calcium), acid-base abnormalities and kidney and liver failure, may cause coma, although there is no evidence that these disorders abolish brainstem reflexes. Judicious clinical judgment is needed to establish a causal link between coma and existing metabolic disorders. IMPORTANT: Metabolic disorders that develop after the setting of coma cannot be considered as the cause and do not interfere with the determination of brain death **(D)**.^(3,35)^

Severe hypothermia may also mimic brain death because the pupillary light reflex is lost at a body temperature ranging from 28 - 32ºC, as well as other brainstem reflexes when the body temperature falls below 28ºC **(C)**.^(36)^ Hypothermia must be corrected ideally to ≥ 35ºC (core blood, rectal, bladder or esophageal temperature) **(D)**^(3,36-38)^ before initiating the neurological examination of patients with suspected brain death **(D)**.

Severe hypotension, independently of its etiology, might cause coma. Ideally the clinical examination for brain death determination should be performed with MAP ≥ 60mmHg or SAP ≥ 100mmHg. The correction of hypotension with fluid infusion and/or vasopressors is adequate **(D)**.^(3)^

The locked-in syndrome, high spinal cord injury and the effects of neuromuscular blocking agents and paralyzing toxins should be considered in the differential diagnosis of motor unresponsiveness **(C)**.^(39)^ However, none of these conditions fulfill the criteria for starting the brain death protocol.

**Recommendation:** Exclude the action of CNS depressants and neuromuscular blocking agents, MAP < 60mmHg or SAP < 100mmHg or body temperature < 35ºC to establish a diagnosis of brain death **(D)**. **Strong Recommendation**.

**Recommendation:** Electrolyte abnormalities that develop after the setting of unresponsive coma do not interfere with the determination of brain death **(D)**. **Strong Recommendation**.

### 11. How to exclude and evaluate the use of central nervous depressants during assessment of patients with suspected brain death?

**Comment:** Brain dead individuals almost always exhibit hypotension, hypothermia, a low metabolic rate and reduced tissue perfusion, resulting in impaired and unpredictable drug metabolism and/or elimination **(D)**.^(40)^ In addition, many CNS depressants have pharmacologically active metabolites with half-lives that are much longer than the parent drug. As a result, the drug effects are more accentuated and last longer than those in healthy individuals, especially when liver or kidney failure is present **(D)**.^(36,41-44)^ CNS depressants should be assessed based on the patient's medical records and, whenever possible, measurement of the drug serum levels. For cases with a known previous history of intoxication, the administration of doses larger than the recommended ones or prolonged administration via continuous infusion, and the drug serum level cannot be measured. Wijdicks et al. suggest waiting a period equivalent to 4 to 5 times the drug half-life before beginning to evaluate brain death in patients with normal liver and kidney function who are not subjected to therapeutic hypothermia, which might delay drug elimination **(D)**.^(41)^ However, it should be noted that this is a private suggestion with a more robust degree of evidence and attuned to the reality of countries in which auxiliary confirmatory tests are not mandatory. Because the law in Brazil requires the performance of confirmatory tests, we might safely set an interval equal to 4 to 5 times the drug half-life for patients without liver and kidney failure and not subjected to therapeutic hypothermia, and preferentially choose tests that are capable of assessing cerebral blood flow, which is not affected by CNS depressants. When intravenous barbiturates are used, cerebral blood flow imaging is mandatory because this type of drug strongly affects the brain metabolism and electrical activity. In patients with unresponsive coma who previously used drugs with potential CNS depressant activity but unlikely to cause unresponsive and areflexic coma at usual therapeutic doses (for example, enteral phenobarbital, phenytoin, clonidine, dexmedetomidine and morphine), these drugs cannot be considered as the cause of coma, and the start of the protocol for brain death diagnosis should not be delayed. By contrast, in patients with kidney or liver failure or who are subjected to therapeutic hypothermia, the pharmacokinetics of drugs might become considerably altered, with consequent prolongation of their effects. Under such circumstances, the interval between drug discontinuation and the start of the diagnostic protocol should be assessed on a case-by-case basis considering the severity of the liver and/or kidney dysfunction, the drug serum level and cerebral blood flow imaging, the latter being mandatory in this case. Electroencephalograms should be avoided in cases with a history of CNS depressant use, induced hypothermia, metabolic disorders or impaired metabolism and excretion of CNS depressants because these conditions might interfere with the test to the point of showing electrical inactivity in patients with preserved cerebral blood flow and consequently resulting in a false-positive diagnosis of brain death **(D)**.^(40-42)^ Serum levels below the therapeutic range allow the exclusion of CNS depressants as the cause of coma; however, this test is not usually available in Brazilian emergency services and thus is seldom used **(D)**.^(40,41)^ While antagonists of benzodiazepines and opioids, such as flumazenil and naloxone, respectively, may be used, they are only able to reveal the presence of brain activity that has been previously masked by CNS depressants, and they do not exclude potential effects of the latter when the patient remains in areflexic coma following their administration **(C)**.^(13)^ Attention should also be paid to neuromuscular blocking agents, even when administered at their usual doses, because they cause paralysis and apnea and thus confound the neurological examination; the orientations given for CNS depressants also apply in this case (Table 2).

**Table 2:** Main central nervous system depressants and interval from discontinuation to the start of the determination of brain death

Agent	Half-life	Interval (if single or intermittent dose)	Interval (if continuous infusion)	Interval (liver/kidney failure)
Midazolam	2 hours	6 hours	10 hours	Individualized
Fentanyl	2 hours	6 hours	10 hours	Individualized
Thiopental	12 hours	36 hours	60 hours	Individualized
Halothane	15 minutes	45 minutes	1 hour and 15 minutes	Individualized
Isoflurane	10 minutes	30 minutes	50 minutes	Individualized
Sevoflurane	12 minutes	36 minutes	1 hour	Individualized
Succinylcholine	10 minutes	30 minutes	50 minutes	Individualized
Pancuronium	2 hours	6 hours	10 hours	Individualized
Atracurium	20 minutes	1 hour	1 hour and 40 minutes	Individualized
Cisatracurium	22 minutes	1 hour and 6 minutes	1 hour and 50 minutes	Individualized
Vecuronium	1 hour and 5 minutes	3 hours and 15 minutes	5 hours and 25 minutes	Individualized
Rocuronium	1 hours	3 hours	5 hours	Individualized
Etomidate	3 hours	9 hours	15 hours	Individualized
Ketamine	2 hours and 30 minutes	7 hours and 30 minutes	12 hours and 30 minutes	Individualized
Propofol	2 hours	6 hours	10 hours	Individualized

Half-life - half-life time; intermittent dose - less than 4 doses/24 hours; continuous infusion - continuous infusion or more than 3 intermittent doses/24 hours. - If intermittent administration: interval of three times the half-life. Use of blood flow imaging test preferred. - If continuous infusion administration: interval of five times the half-life. Use of blood flow imaging test preferred. - In liver and/or kidney failure: determine the interval on a case-by-case basis, considering the severity of the abnormalities, discussing the case with the intensivist and with the on-call doctor of the Organ Procurement Organization/Center for Notification, Procurement and Allocation of Organs. In these cases, the blood flow imaging test is mandatory. - In the case of an intravenous barbiturate, always perform the blood flow imaging test. - The cause of unresponsive and areflexic coma should not be related to CNS depressants that are unlikely to cause areflexic coma when used at usual therapeutic doses. Examples: enteral phenobarbital, phenytoin, clonidine, dexmedetomidine, morphine.

**Recommendation:** Rule out action of CNS depressants prior to the determination of brain death as follows **(D)**. **Strong Recommendation**:

- Unresponsive areflexic coma must not be attributed to CNS depressants without the potential to cause areflexic coma when administered at their usual therapeutic doses (e.g., enteral phenobarbital, phenytoin, clonidine, dexmedetomidine and morphine).
- Wait a period equivalent to four to five times the half-life of CNS depressants after their discontinuation, provided these agents were administered in continuous infusion and at their usual therapeutic doses; cerebral blood flow imaging is advisable. In the case of intravenous barbiturates, cerebral flow imaging is mandatory.
- For patients with liver or kidney failure or after induced therapeutic hypothermia, estimate the interval between drug discontinuation and initiation of the brain death protocol, taking into account the severity of liver and/or kidney dysfunction. The serum levels of CNS depressants may be considered, and **cerebral blood flow imaging is mandatory as an auxiliary test.**

**Recommendation:** Any decision that has been made based on the use of CNS depressants should be recorded in complete detail with due justification of the patient's medical records **(D)**. **Weak recommendation**.

### 12. Which auxiliary tests can be used for the diagnosis of brain death? Are there circumstances that require certain tests?

**Comment:** A technically scientific diagnosis of brain death is established based on the clinical examination **(D)**.^(3)^ However, when a neurological examination cannot be performed due to technical problems (e.g., eye injuries, apnea test not possible due to hypoxemia) or potential interference by confounding factors such as hypothermia, metabolic disorders and CNS depressant use, or due to legal reasons as in Brazil, an imaging test demonstrating an absence of cerebral blood flow, electrical activity or metabolic and cephalic activity is required **(D)**.^(3,23,45)^ The ideal auxiliary test should have adequate sensitivity but mainly 100% specificity, which indicates that there will be no cases of patients presenting any evidence of brain or brainstem activity during the clinical examination for which the imaging test shows an absence of cerebral blood flow or of electrical or metabolic activity (false positive). Safety and the immediate availability of the imaging test are also desirable characteristics. SAP ≥ 100mmHg and MAP ≥ 60mmHg are required for all imaging tests to avoid false-positive results **(D)**.^(46,47)^

Several tests are accepted internationally for the determination of brain death **(D)**.^(3,32,45-48)^ **Cerebral angiography**, which investigates the presence of blood flow in the intracranial portion of the internal carotid and vertebral arteries, is considered the reference standard for the test comparison. However, it has some disadvantages, such as a need to move the patient outside the ICU, the use of potentially nephrotoxic contrast agents and arterial puncture **(D)**.^(45,46)^ **Transcranial Doppler ultrasonography** investigates the presence of blood flow in the intracranial internal carotid, middle cerebral, vertebral and basilar arteries. Although this test should be performed by a professional with a high level of training, it has the following advantages: bedside availability, noninvasive and does not require the use of contrast medium **(D)**.^(45,46)^ **Cerebral scintigraphy** is also a widely accepted cerebral blood flow test; it assesses brain perfusion based on parenchymal uptake of the radionuclide technetium. This test does not require the use of iodinated contrast, is easy to interpret and exhibits high concordance with cerebral angiography **(D)**.^(45,46)^ As a significant advantage, none of these cerebral blood flow tests are influenced by CNS depressants, hypothermia or metabolic disorders; therefore, they are recommended for patients under any of these described circumstances over tests that assess brain electrical activity. The main limitation of these tests is that they might demonstrate cerebral blood flow in patients with some degree of skull opening, such as children under 1 year of age, individuals with open head injuries or after extensive craniotomy. Under such circumstances, tests assessing electrical activity might be preferred, even though a demonstration of no cerebral blood flow provides definitive confirmation **(D)**.^(45,46)^ **Encephalography** is a widely used imaging test that investigates the presence of electrical activity. Its advantages are performance at the bedside, no requirement for contrast medium and wide availability. Its main disadvantage is that it might demonstrate an absence of electrical activity in the presence of confounding factors, namely, severe metabolic disorders, hypothermia and CNS depressant effects **(D)**.^(45,46)^ In this case, cerebral blood flow imaging must be performed. In this context, it is worth underscoring that continuous administration of barbiturates has a cumulative effect in which electrical activity might remain absent on electroencephalography for several hours after discontinuation **(D)**.^(3,23,45-48)^

Several other tests have been assessed, including **computed tomography angiography** (CTA), a previously validated cerebral blood flow test that is currently used in some countries and gaining increasing acceptance worldwide. This test is attractive because it is available at most medium- and large-sized hospitals, is easy to perform, does not require invasive puncture and employs a lower amount of iodinated contrast medium compared to conventional angiography. While most studies have assessed individuals with a confirmed diagnosis of brain death only **(B)**,^(49-51)^ Dupas et al. included a control group, i.e., coma patients still exhibiting evidence of brain electrical activity, and found that the method had 100% specificity **(B).**^(52)^ There are no uniform international radiological criteria for the interpretation of CTA; however, the *Société Française de Radiologie* published guidelines based on a so-called "four point scale" that assesses opacification of the middle cerebral arteries and internal cerebral veins with 85% sensitivity **(D).**^(53)^ Two recent systematic reviews concluded that CTA might be used as an auxiliary to clinical examination for the diagnosis of brain death **(B)**.^(54,55)^

**Evoked potentials**, which assess brain electrical activity, are of limited use because they investigate specific neural pathways, even when including somatosensory and auditory evoked potentials, which assess the electrical response to stimulation of the median and vestibulocochlear nerve, respectively. The principle underlying the evoked potentials is alien to the concept of and rationale for the integral and global assessment of the brain function required for an accurate diagnosis of brain death. The test might evidence the absence of electrical signals in lesions proximal to the investigated pathways, even though other areas might be preserved from an anatomical and functional perspective. Few studies have investigated evoked potentials in coma patients with severe brain injury but not fulfilling the clinical criteria for brain death, which does not allow an accurate assessment of the specificity of the method. Similarly to electroencephalograms, evoked potentials can also provide false-positive results in the presence of hypothermia, metabolic disorders or the use of CNS depressants **(B)**.^(56-58)^

**Intracranial pressure monitoring** is indicated based on physiological reasons, but it has been assessed in only a few observational studies with a small number of patients. When the intracranial pressure remains above SAP continuously for at least 20 minutes, the test is considered to be positive. One further limitation of this method is the technical difficulty inherent to the available measurement techniques, which provide values with reduced accuracy. Therefore, intracranial pressure monitoring should not be used for the diagnosis of brain death **(C)**.^(59-61)^

**Jugular venous oxygen saturation monitoring** has a physiological rationale, namely, the drop in the oxygen extraction rate that occurs at the time of brain death. This parameter was assessed in a single prospective observational study that evaluated a central venous/jugular oxygen saturation ratio < 1 as predictor of brain death in a sample of 118 individuals with a clinical diagnosis of brain death and 152 head injury survivors. The test had 96.6% sensitivity and 99.3% specificity for the diagnosis of brain death. Electroencephalography was the imaging method used as a reference for comparison. Jugular venous oxygen saturation monitoring is limited by technical difficulties related to the position of the catheter, and the results are influenced by the PaO_2_ level. Therefore, it should not be used for the diagnosis of brain death **(B)**.^(62)^

**Recommendation:** The preferred imaging tests for the diagnosis of brain death are cerebral angiography, transcranial Doppler ultrasonography, cerebral scintigraphy and electroencephalogram **(D)**. **StrongRecommendation**.

**Recommendation:** Intracranial pressure and jugular venous oxygen saturation should not be used as imaging tests for the diagnosis of brain death. **(C)**. **Strong Recommendation**.

**Recommendation:** In cases with severe metabolic disorders, hypothermia and the use of CNS depressants, cerebral blood flow tests are indicated: cerebral angiography, transcranial Doppler ultrasonography and cerebral scintigraphy **(D)**. **Strong Recommendation**.

**Recommendation:** Computed tomography angiography may be used as an auxiliary means for the diagnosis of brain death at institutions with a standardized protocol for interpretation of the results, such as the *Société Française de Radiologie*'s "four point scale" **(B)**. **Weak Recommendation**.

**Recommendation:** In patients with some degree of skull opening, such as children under 1 year of age, individuals with open head injuries or after an extensive craniotomy, electroencephalography might be preferred, but only residual blood flow has been demonstrated using other methods **(D)**. **Strong Recommendation**.

### 13. In situations such as severe facial trauma, otorrhagia, eye agenesis and high cervical spine injury, which preclude the performance of a portion of the clinical examination, is it possible to establish a diagnosis of brain death?

**Comment:** In Brazil, the determination of brain death is based on a confirmed irreversible loss of all brainstem functions, as established on the clinical examination and by the apnea test, whereas an auxiliary test might be performed as an additional safety measure and to provide documented proof of the patient's status. Any hindrance to the performance of some part of the brainstem function assessment might raise doubts regarding the diagnosis of brain death and, concomitantly, represent a situation of ethical-legal non-compliance with the stipulations of law no. 9,434 and CFM resolution 1,480 **(D)**.^(18,19)^

There are no data in the literature or derived from daily clinical practice to contraindicate continuation of the process of brain death determination when one of the brainstem reflexes cannot be evaluated, provided all other findings on the clinical examination are compatible with brain death **(D)**.

The results of 18 years of experience since it was passed show that resolution no. 1,480 requires modifications **(D)**.^(19)^ In 2011, the CFM Brain Death Technical Board passed a new resolution on brain death determination that has not yet been enforced. Article 3, paragraph 4 of this new resolution establishes that "in the presence of congenital or acquired structural abnormalities hindering the assessment of the reflexes mentioned in the heading of this article, and provided all other findings on clinical examination confirm the status of brain death, due justification for the aforementioned impossibility should be recorded in the [patient's] medical records and [the process of brain death] determination should continue". Only once the new resolution is in force will there will be legal and ethical grounds to continue the process of brain death determination under the aforementioned circumstances.

**Recommendation:** According to CFM resolution no. 1,480 and law no. 9,434, the examination of brain death cannot continue when it is not possible to assess all brainstem reflexes **(D)**. **Strong Recommendation**.

### 14. Who is responsible for filling and signing the death certificate? What time of death should be recorded in the death certificate? Can therapeutic support be discontinued after a diagnosis of brain death is established?

**Comment:** Only doctors can issue a death certificate **(D)**,^(63)^ with the exception of cases of natural death in a place where no doctor is available, as stipulated in law 12,842/2013 **(D)**,^(63)^ which regulates the practice of medicine.

The death certificate should be filled by the doctor who confirmed the occurrence of death, according to a CFM resolution **(D)**.^(64)^ In the case of brain-dead individuals, a brain death certificate is first issued,^(18)^ and when natural death occurs, the death certificate is issued by the doctor who cared for the patient or by a substitute or on-call doctor if the former is not available **(D)**.^(64,65)^ "Death Verification Services are institutions with the aim to determine that death has actually occurred, as well as its cause - when death was natural and there is no suspicion of violence - in cases of death without previous medical care, or in cases in which medical care was provided but death occurred due to an ill-defined condition". Therefore, wherever a Death Verification Service is available, it may be called in when the doctor is unable to correlate death to the clinical condition of the patient, as recorded in his/her medical records or institutional medical forms **(D)**.^(65)^

In cases of unnatural death, also known as death due to external causes (homicide, accidents, suicide and suspicious deaths), at places where there is a *Instituto Médico Legal* (IML) unit, the death certificate is issued by the coroner **(D)**,^(64,65)^ and the attending doctor should complete the Cadaver Referral to the IML Form (Guia de Encaminhamento de Cadáver ao IML). In places where no IML unit is available, the death certificate is issued by a local doctor who is appointed as the ad hoc coroner by legal or police authorities **(D)**.^(64,65)^

The date and time of death recorded in the death certificate should be those corresponding to the determination of brain death, according to CFM resolution no. 1,826/2007 **(D)**.^(66)^

Brain death is equivalent to death. Therefore, from an ethical and legal perspective, once brain death is diagnosed in a non-donor, the doctor must discontinue all support procedures that artificially sustain the function of vital organs. In this case, discontinuation of life support does not characterize orthothanasia, euthanasia or any threat to life because the subject is not a terminal patient but a cadaver. Resolution no. 1,827/2007 states that "discontinuation of therapeutic support procedures after brain death was determined in a non-donor is legal and ethical" **(D)**.^(66)^ Here the term "non-donor" encompasses not only cases due to family refusal but also to medical contraindication and/or to administrative/logistic problems. Doctors should communicate the patient's death to the family or legal representatives in a clear and detailed manner and enter in the patient's medical records the date and time of the communication, as well as the names of the individuals who were present. Maintenance of therapeutic support for brain-dead non-donors may be considered in the case of pregnant women with a living fetus, in which case the corresponding decisions should be made by an obstetrician.

**Recommendation:** In cases of death due to natural causes, the death certificate must be completed and signed by the doctor who provided care to the patient and determined the presence of brain death or by a substitute **(D)**. **Strong Recommendation**.

**Recommendation:** In cases of death due to unnatural causes, the death certificate must be completed and signed by a coroner, while the attending physician, a substitute or an on-call doctor must provide all the relevant information related to the case in point **(D)**. **Strong Recommendation**.

**Recommendation:** The date and time of death recorded in the death certificate should be those corresponding to the last procedure for the determination of brain death** (D)**. **Strong Recommendation**.

**Recommendation:** All therapeutic support should be discontinued after brain death is determined in a non-donor and this information has been communicated to the family with an explanation for the patient's death **(D)**. **Strong Recommendation**.

## PART 3: CRITERIA FOR POTENTIAL DONOR SELECTION

### 15. How should the clinical and laboratory assessment of potential organ and tissue donors be organized and performed?

**Comment:** Any risk of the transmission of infectious or neoplastic diseases through organ or tissue transplantation should be completely eliminated. The risks associated with the procedure should always be considered in relation to the high risk of the death of patients on the transplant waiting list. The increasing number of individuals on waiting lists and of needed transplant organs has led transplantation teams to use organs from donors with expanded criteria, with satisfactory outcomes, but with a higher potential for complications such as disease transmission. All procedures needed to gather the clinical and laboratory data to determine a minimum risk to the recipient of the organs and tissues used for transplantation should be performed. The time available to assess potentially deceased donors is usually quite short, particularly in the case of solid organ donors.^(17,67,68)^ Consequently, well-structured approaches are required to be applied by health care providers with direct participation in the donation-transplantation process. To ensure the safety and quality of the donation-transplantation process, which includes the clinical assessment of potential donors and performance of auxiliary tests, it is advisable to appoint a duly trained professional to oversee the entire process **(D)**.^(17)^

The assessment comprises the following steps: (1) clinical history (analysis of the patient's medical records and interviews with relatives), (2) physical examination including anthropometric measurements, (3) auxiliary tests and (4) inventory during organ removal surgery.

1. *Clinical history:* aims at ruling out transmissible (infectious and neoplastic) diseases in the donor, in addition to determining the functional status of the organs to be harvested and transplanted. For this purpose, the donor's clinical history should be carefully reviewed to guide selection of the necessary auxiliary tests **(D)**.^(68)^

A careful review of the potential donor's medical records allows information to be gathered concerning the cause of death, current disease, past pathological history, treatments administered and intercurrent events **(D)**.^(68)^

A detailed clinical history serves to confirm the donor's past medical history (with an emphasis on neoplastic and infectious diseases), social habits (diet, alcohol and/or illegal drug use, smoking), sexual behavior, occurrence of menstrual irregularity after pregnancy (choriocarcinoma), admission/stay at institutions (arrests/psychiatric hospitals), origin and geographical provenance **(D)**.^(68)^

2. *Physical examination and anthropometric measurements:* allow the detection of clinical conditions that might contraindicate the donation and/or suggest laboratory tests to dispel doubts about the eligibility of the donor, in addition to assessing the compatibility between the size of the transplant organ and the biotype of the recipient. The features to be explored during physical examination include scars/puncture wounds due to illegal drug use, trauma injuries, tattoos, geographic characteristics, masses/enlarged lymph nodes, skin neoplasms and scars derived from past surgical interventions.

The anthropometric variables to be assessed are as follows:

- Body weight and height: all donors
  - Pediatric kidney donor: > 15kg, separate removal; < 15kg, en bloc removal of the kidneys.
  - Liver donor: approximately 10 - 20% variation, with a donor weight and graft weight to recipient weight ratio of 1% - the latter especially in the case of children.
  - Pancreas donor: acceptable when the body weight is between 30 and 90kg.
  - Heart donor: < 20% lower weight.
- Chest circumference at the level of the nipple: lung donors.

3. *Auxiliary tests:* allow monitoring of clinical parameters during donor maintenance to detect organ dysfunctions and transmissible diseases and provide guidance to prioritize possible recipients included in the waiting list according to their blood type.

- Periodic biochemical testing every 24 hours to attain normal physiological parameters and ensure adequate functioning of the transplant organs **(D)**.^(69,70)^
- Particular tests should be performed according to the organs to be transplanted: Heart donor - creatine kinase MB isoenzyme (CK-MB) and/or troponin every 24 hours, electrocardiogram and echocardiogram; cardiac catheterization may be considered for donors >45 years old. Liver donor - aspartate aminotransferase (AST), alanine aminotransferase (ALT) and bilirubin at least every 24 hours. Kidney donor - urea and creatinine (Cr) every 24 hours and urinalysis. Pancreas donor - amylase and blood glucose every 24 hours. Lung donor - arterial blood gases with FiO_2_ at 100% and chest radiography **(D)**.^(68,69,71)^
- The presence of transmissible diseases should be eliminated by performing serologic tests for Chagas disease, anti-toxoplasma antibodies, Venereal Disease Research Laboratory (VDRL, when positive, the fluorescent treponemal antibody absorption test (FTA-ABS) should be performed), anti-human immunodeficiency virus (HIV) antibodies, anti-human T lymphotropic virus (HTLV) 1 and 2 antibodies, surface antigen of hepatitis B virus (HBsAg), hepatitis B core antibodies (anti-HBc), hepatitis B surface antibodies (anti-HBs), hepatitis C antibodies (anti-HCV), cytomegalovirus antibodies (anti-CMV), Epstein-Barr virus antibodies (anti-EBV) and serologic tests for malaria in endemic areas (North Brazil).
- Two blood cultures and one urine culture should be performed for all potential donors. Cultures with samples collected from other body sites should be performed in the case of suspected infection; the results must be supplied to the transplantation teams/transplant centers **(D)**.^(71,72)^
- Tumor markers: see question 21.

4. *Surgical inventory during organ removal:* the chest and abdominal organs should be examined during removal surgery to detect potentially hidden tumors or pathological lymph nodes. The kidneys and liver should be carefully examined due to the high numbers of tumors found in kidneys after removal **(D)**.^(73,74)^

**Recommendation:** Perform a complete clinical history, including a past pathological history and careful physical examination, order auxiliary tests (Table 3) and perform a surgical inventory during organ removal **(D)**. **Strong Recommendation**.

**Table 3:** Examinations to be solicited for the evaluation of the potential donor.

Assess	Examination
Blood type	ABO group
Serology	Anti-HIV, HTLV-I and II, HBsAG, anti-HBc, anti-HBS, anti-HCV, CMV*, Chagas disease, toxoplasmosis* and VDRL
Hematology	Blood count and platelets
Electrolytes	Sodium, potassium, magnesium and phosphorus
Lung donor	Arterial gas and chest radiograph
Heart donor	Troponin, CK-MB, electrocardiogram, echocardiography and cardiac catheterization**
Kidney donor	Urea, creatinine and urinalysis
Liver donor	GOT, GPT, gamma-GT and bilirubin
Pancreas donor	Amylase and blood glucose
Infections	Two blood cultures and cultures of materials from body sites with suspected infection
Neoplasia	β-hCG in female donors of reproductive age

HTLV - human T-lymphotropic virus; HBsAG - hepatitis B virus surface antigen; anti-HBc - hepatitis B core antibody; anti-HBS - antibodies against hepatitis B surface antigen; anti-HCV - antibodies against hepatitis C virus; CMV - cytomegalovirus; VDRL - Venereal Disease Research Laboratory; CK-MB - creatine kinase MB isoenzyme; GOT - glutamic-oxalacetic transaminase; GPT - glutamic-pyruvic transaminase; gamma-GT - gamma-glutamyltransferase; β-hCG - beta-human chorionic gonadotropin.

* Results may be obtained after transplantation.

** For patients older than 45 years.

**Recommendation:** Enter all findings on the complete clinical assessment in the medical records of the potential donor **(D)**. **Strong Recommendation**.

### 16. Which organs and tissues harvested from brain-dead donors can be donated?

**Comment:** Brain-dead deceased donors are the main source of transplant organs and tissues **(D)**.^(75)^ According to the Brazilian Ministry of Health ordinance no. 2,600, from September 21, 2006 **(D)**,^(76)^ the organs that can be donated and individually used for transplantation are the heart, lungs, kidneys, liver, pancreas and intestine **(B)**.^(69,76-78)^ Multi-organ transplantation can also be performed, which involves joint donation and transplantation of the liver, pancreas, stomach, duodenum and small intestine into a single recipient; other possibilities are kidney-pancreas and liver-kidney transplantation. Tissues that can be donated for transplantation are the cornea, sclera, skin, bone, cartilage, tendon, meniscus, muscle fascia, heart valves, pericardium and blood vessels **(B)**.^(69,76-78)^ Hematopoietic stem cells retrieved from the bone marrow, peripheral blood and the umbilical cord/placenta can be donated by living donors. There are reports in the literature of limb **(C)**,^(79,80)^ face **(C)**,^(81)^ larynx and trachea **(C)**^(82)^ transplants, which are not performed in Brazil.

**Recommendation:** Organs that can be donated by brain-dead deceased donors include the heart, lungs, kidneys, liver, pancreas and intestine **(B)**. **Strong Recommendation**.

**Recommendation:** Tissues that can be donated by deceased donors are the corneas, sclera, skin, bone, cartilage, tendon, meniscus, muscle fascia, heart valves and blood vessels **(B)**. **Strong Recommendation**.

### 17. What characterizes the expanded criteria donor?

**Comment:** Several terms are used to designate donors that barely meet the selection criteria, such as suboptimal, unfit, high-risk, marginal, borderline and expanded criteria donors. The terms "high risk", "marginal" and "expanded criteria" donors are the most widely used **(D)**.^(83)^ There is no universal definition of marginal or expanded criteria donors (ECDs). However, the presence of some conditions associated with shortened survival, reduced graft function or the risk of disease transmission has been used to characterize organs as of "marginal" quality **(D)**.^(83-85)^

The characteristics of marginal donors are as follows: **(D)**:^(83-85)^

**- Relative to graft function**: higher short-term morbidity (delayed graft function or primary graft nonfunction) and shorter graft survival. These events might be associated with the donor's age, past pathological history, anthropometric measurements, cause of death, previous function of the organ to be donated, anatomical abnormalities, intoxications and poisonings, hemodynamic instability, prolonged ischemia time and donation after circulatory death **(D)**.^(86-93)^
**- Relative to disease transmission**: infections and neoplasias.

The use of marginal donors is only justified when the life expectancy after transplantation is higher compared with conventional clinical treatment. Under borderline circumstances, the decision to transplant organs is made by the transplantation team with the informed consent of the recipient. The organs must be removed, and if they are not used in the same Brazilian state, then they should be offered to the National Transplant Center for allocation to other states.

**Recommendation:** Marginal or expanded criteria donors are those presenting clinical conditions that might reduce graft survival, impair its function or are at high risk of disease transmission **(D)**. **Strong Recommendation**.

**Recommendation:** The use of marginal donors is only justified when the life expectancy after transplantation is higher compared with conventional clinical treatment **(D)**. **Strong Recommendation**.

### 18. What is the accepted age range for multiple organ and tissue donors?

**Comment:** Any brain dead individual may be considered a potential donor independent of his/her age **(D)**.^(94)^ In Brazil, the minimum age for the determination of brain death and characterization as an organ donor is 7 days **(D)**.^(18,76)^ There is no maximum age for donation; however, comorbidities that develop together with aging make donation less acceptable **(D)**.^(95)^

Kidney graft function and survival are impaired when donors are greater than 60 years old (**D**).^(95)^ Expanded criteria kidneys are those that are harvested from donors greater than 70 years old with no additional risk factors and from donors aged 60 to 70 years old with a history of diabetes, systemic arterial hypertension, significant proteinuria (over 1 g/24 hours) and signs of hypertension- or diabetes-related target organ injury. Kidneys from such donors are associated with a higher risk of death and graft loss, especially when transplanted into recipients under 60 years of age **(D)**^(96,97)^ **(B)**.^(98)^

Relative to the liver, age alone does not define contraindications; however, graft function and survival might be impaired when donors are more than 60 years old **(D)**,^(95)^ and livers from donors greater than 90 years old should not be used **(D)**.^(99)^ Donation is also contraindicated in the case of donors over 65 years old who present hepatic steatosis, gamma-glutamyltransferase (gamma-GT) elevated to more than three times its normal value, a prothrombin time below 40% or a platelet count less than 60,000/mm^3^ **(D)**.^(100)^

Despite the higher mortality of patients who received hearts from donors greater than 64 years old, age does not represent an absolute contraindication to heart donation **(D)**.^(101)^ The age limit depends on the local stipulations of the protocol and the condition of the recipient **(D)**.^(95)^ The performance of coronary angiography has been suggested to eliminate the possibility of coronary artery disease in heart donors over 45 years old **(D)**.^(96)^ The National Transplantation System technical regulations leave decision-making on maximum age to the transplantation teams **(D)**.^(76)^

Ideally, lung donors should be under 55 years of age **(D)**,^(102)^ with a maximum age of 60 years old according to the aforementioned technical regulation **(D)**.^(76)^ However, as a function of the condition of the donor and the protocol applied by the transplantation team, **(D)**^(76)^ the maximum age might rise to 65 years old.

For simultaneous pancreas-kidney transplantation, the donor's age should be 18 to 45 years old. This restriction is used to control the distribution of organs because the waiting list for kidney transplants alone is much longer than the one for kidney-pancreas transplantation. According to the aforementioned technical regulations, the minimum and maximum age for pancreas transplant alone is 7 days and 50 years old, respectively **(D)**.^(76)^ However, according to some reports in the literature, the ideal age range for pancreas donors is 10 to 40 years old **(D)**.^(103)^

Relative to tissue donation, there are age restrictions for some tissues according to the stipulations of individual protocols **(D)**.^(95)^ In Brazil, the age range for cornea donors is 2 to 70 years old, for tendon donors is 18 to 55 years old and for osteochondral tissue donors is 15 to 45 years old **(D)**.^(76)^

**Recommendation:** For recipients under 45 years old, the ideal age of deceased donors is as follows: kidneys up to 60 years old; liver up to 60 years old; kidney-pancreas 18 to 45 years old; pancreas 7 days to 50 years old; heart up to 45 years old; and lungs 60 to 65 years old **(D)**. **Weak Recommendation**.

**Recommendation:** Individuals over 80 years old might also be considered as organ donors **(D)**. **Weak Recommendation**.

### 19. What are the absolute contraindications for organ donation for transplantation?

**Comment:** The use of organs from a potential donor is absolutely contraindicated when the risk of disease transmission is superior to the possible benefits to the potential recipients. The main absolute contraindications are related to the transmission of some infectious^(72)^ and neoplastic^(104)^ conditions.

1. *Infectious conditions that absolutely disqualify a potential donor:* Infection with HIV was considered an absolute contraindication to organ donation until very recently. There are reports of HIV transmission to recipients from seronegative organ donors.^(104)^ A case series of transplantation involving HIV-positive deceased donors and HIV-positive recipients from Africa was reported, with satisfactory outcomes at the 1- and 5-year follow-ups, similar to those in other populations.^(104,105)^ Although this approach was suggested as an option to overcome the shortage of transplant organs in areas with a high prevalence of HIV infection, the effects of the transmission of different viruses and the long-term outcomes of the patients are not known.

The incidence of human T-cell lymphotrophic virus type-I or type-II (HTLV-I/II) infection in non-endemic areas is low (less than 1%), and the rate of false-positive cases is high. Only HTLV-I is associated with adult T-cell leukemia/lymphoma (ATLL) and tropical spastic paraparesis. In infected individuals, the lifetime risk of developing ATLL ranges from 2% to 5% and of developing paraparesis ranges from 1% to 2%. Although there are reports of disease transmission to six organ recipients from four donors, only one of whom had confirmed HTLV infection, no case of recipient death by infection with HTLV has yet been reported **(D)**.^(73,74,106,107)^ Thus, some countries with a low prevalence of HTLV infection (United States and England) and an insufficient number of donors to meet the increasing demand for transplant organs allow the performance of transplants without previous knowledge of the HTLV status of the donor **(D)**.^(73,74,106,107)^

Cases of death by transmission, from organ donors to recipients, of rabies,^(108,109)^ West Nile virus,^(110)^ lymphocytic choriomeningitis and *Cryptococcus neoformans*^((111)^ have been reported. The use of organs for transplantation from donors with any of these conditions or encephalitis of unknown cause is contraindicated.

Due to the window of time that permits the detection of antibodies against some viruses, there is an interval between the onset of infection and its laboratory detection (HIV: average of 22 days; HCV: average of 60 days). The risk of transmission of these diseases is considered to be lower than the risk of death exhibited by patients waiting for transplant organs. The risk of disease transmission during the window period is higher for high-risk donors (Table 4). Nucleic acid testing (NAT) reduces the window period (from 22 to 9 days for HIV and from 60 to 7 days for HCV), with a consequent decreased risk of transmission.^(112-116)^

**Table 4:** Factors associated with an increased risk for recent infection with HIV, hepatitis B virus or hepatitis C virus

**Risk factors for HIV, HBV or HCV**	
1.	Individual who had sexual intercourse with a partner with confirmed or suspected infection with HIV, HBV or HCV in the past 12 months
2.	A man who had sex with a man in the past 12 months
3.	A woman who had sex with a man with a history of having sex with men in the past 12 months
4.	An individual who exchanged sex for money or drugs in the past 12 months
5.	An individual who had sexual intercourse with an injection drug user in the past 12 months
6.	A child under 18 months old from a mother infected with or at high risk of infection with HIV, HBV or HCV
7.	A child breastfed in the past 12 months by a mother infected with or at high risk of infection with HIV, HBV or HCV
8.	An individual with a history of injection drug use (IV, IM or SC)
9.	A person with a history of time in jail or a juvenile correctional facility for more than 72 hours in the past 12 months
10.	Individuals diagnosed with or treated for syphilis, gonorrhea, chlamydia or genital ulcers in the past 12 months
**High risk for HCV only**	
1.	An individual with a history of hemodialysis in the past 12 months

HIV - human immunodeficiency virus; HBV - hepatitis B virus; HCV - hepatitis C virus; IV - intravenous IM - intramuscular; SC - subcutaneous.

2. *Neoplastic conditions that absolutely disqualify a potential donor:* A recent history of or an active malignant neoplasm, excluding tumors with a low risk of transmission, such as ^(117)^ skin basal cell carcinoma, cervical carcinoma *in situ* and primary CNS tumors (excluding high-grade medulloblastoma, glioblastoma and astrocytoma) **(C)**.^(118,119)^ Only two cases of transmission of primary CNS tumors have been reported, with both patients having glioblastoma multiforme **(C)**.^(120,121)^ Decision-making is difficult in cases with old or theoretically cured neoplasms. There are reports of the occurrence of breast cancer **(C)**^(122)^ and melanoma **(C)**^(123)^ in organ recipients aged 8 and 32 years, respectively, after the donors were declared to be cured. Therefore, decision-making should be based on the disease-free period, tumor histology and stage **(C)**.^(124,125)^

Clinically uncontrolled sepsis is a contraindication to organ donation. Potential donors with sepsis, but hemodynamically stable and/or undergoing vasopressor tapering, may donate organs. All blood culture results should be verified and communicated to the transplantation center.^(126,127)^

**Recommendation:** When donors are at a high risk of transmission of infectious viral diseases, the recipients should be informed and their consent requested. Whenever possible, NAT should also be performed **(D)**. **Strong Recommendation**.

**Recommendation:** The transplantation of organs from donors with the following infectious conditions is contraindicated: HIV infection, positive serology for HTLV-I and II, acute hepatitis, active tuberculosis, malaria, acute viral infections (e.g., rubella, rabies, West Nile virus, adenoviruses, enteroviruses, parvoviruses, viral meningoencephalitis or of unknown origin), cryptococcal meningitis and prion diseases **(C)**. **Strong Recommendation**.

**Recommendation:** Sepsis that is not clinically controlled (e.g.: no or steady dose vasopressor) contraindicates organ donation. Potential donors with sepsis, but hemodynamically stable and/or undergoing vasopressor tapering, may donate organs. All blood culture results should be verified and communicated to the transplantation center **(C)**. **Strong Recommendation**.

**Recommendation:** Organ donation from donors with any malignant neoplasm should be contraindicated, excluding skin carcinoma *in situ*, cervical carcinoma *in situ* and some primary CNS tumors. Primary CNS tumors that represent contraindications to donation are described in table 5 **(C)**. **Strong Recommendation**.

**Table 5:** Brain tumors and organ donation

Group 1 Tumors that are not a contraindication to multiple organ donation	Group 2 Tumors that might not be a contraindication to donation according to circumstances	Group 3 Tumors that are not a contraindication to multiple organ donation
Benign meningioma	Low-grade astrocytoma (Grade II)	Anaplastic astrocytoma (Grade III)
Pituitary adenoma	Gliomatosis cerebri	Glioblastoma multiforme
Acoustic neuroma		Medulloblastoma
Craniopharyngioma		Anaplastic oligodendroglioma (Schmidt C and D)
Pilocytic astrocytoma (Grade I)		Malignant ependymoma
Epidermoid cyst		Pineoblastoma
Third ventricle choroid plexus cyst		Anaplastic and malignant meningioma
Choroid plexus papilloma		Intracranial sarcoma
Hemangioblastoma		Germ cell tumor (except for well-differentiated teratoma)
Ganglion cell tumor		Chordoma
Pineocytoma		Primary lymphoma of the brain
Low-grade oligodendroglioma (Schmidt A and B)		
Ependymoma		
Well-differentiated teratoma		

### 20. How to deal with donors with a history of infection and how to prevent infection transmission?

**Comment:** Infection transmission by donors is rare; however, it is associated with significant morbidity and mortality **(A)**.^(128,129)^ There are reports of bacterial, viral, fungal and parasitic infection transmission via organ transplantation **(A)**.^(130)^ The identification of active or latent infections in donors allows for a better assessment of risk and the establishment of preventive measures. Given the shortage of organs relative to the number of patients on the transplantation waiting lists, the use of marginal organs, including those with potential for the transmission of infections, is a current reality. An accurate estimation of this risk based on adequate epidemiological, clinical and laboratory assessments is essential to minimize negative consequences and improve recipient survival. Assessment and selection are more difficult in the case of deceased donors due to the short time available **(B)**.^(131)^

#### Bacterial infections

The greatest risk of transmission of bacterial infections is posed by donors with bacteremia caused by either Gram-positive cocci or Gram-negative bacilli **(B)**.^(127,132)^ The assessment of donors includes blood culturing at preset times to detect bacteremia in approximately 5% of cases, even in the absence of clinical evidence of infection **(A)**.^(133,134)^ Intervention studies reported a significant reduction of the risk of transmission when donors received antibiotics for at least 48 hours and exhibited clinical improvement and recipients were treated based on the bacteria isolated from the donors for at least 7 days. These studies mainly involved donors with endocarditis or meningitis **(B)**.^(135-137)^ Therefore, blood cultures should be performed for all donors to identify infection, eventually demonstrating no bacteria and guiding the antibiotic regimen administered to the recipient **(B)**.^(127,131)^ In the case of lung donors, respiratory secretion samples should also be collected because evidence of infection by bacteria colonizing the airways of donors have been found in transplant recipients **(B)**.^(138)^ The use of organs that are colonized or infected with multidrug resistant bacteria is a highly relevant and controversial issue. There are no data in the literature providing evidence for or against the use of such organs **(C)**.^(130,139,140)^

The transmission of tuberculosis via transplanted organs has also been reported **(B)**.^(141)^ The infection might have a fatal outcome in recipients, and therefore, organs from donors with known infection must be discarded. The investigation of latent infection in donors, including radiological methods, the tuberculin skin test and the interferon-gamma release assay, is difficult, and a positive result does not necessarily lead to the exclusion of donors; however, it might guide the preventive measures administered to recipients **(C)**.^(142)^

Syphilis is another bacterial infection that may be latent or asymptomatic, and cases of transmission through organ transplantation have been reported. Infection does not represent a contraindication to donation, but recipients should receive appropriate treatment after transplantation **(B)**.^(143)^

**Recommendation:** Collect at least two blood samples for culture from all donors. Culture of respiratory secretions should also be performed for lung donors.** (B)**. **Strong Recommendation**.

**Recommendation:** Urine culture must be performed for kidney donation** (D)**. **Strong Recommendation**.

**Recommendation:** Donation from donors with bacterial infection is not contraindicated provided the donor has undergone efficacious antibiotic therapy, preferably for at least 48 hours **(B)**. **Strong Recommendation**.

**Recommendation:** Recipients should receive antibiotic treatment based on the bacteria isolated from or the antibiotics administered to the donor for at least 7 days **(C)**. **Strong Recommendation**.

**Recommendation:** Donor colonization by multidrug resistant bacteria is not a contraindication to donation, except in the following cases: colonization by bacteria for which there is no efficacious treatment and colonization of the transplant target site (urinary tract for kidney transplants; gastrointestinal tract for liver, pancreas and intestine transplants; airways for lung transplant; and central venous catheter related area for heart transplants) **(C)**. **Weak Recommendation**.

**Recommendation:** Organs from donors with active tuberculosis and without efficacious treatment for at least 2 months should not be used **(C)**. **Strong Recommendation**.

**Recommendation:** Positive serology for syphilis is not a contraindication to donation, but recipients should receive specific treatment after transplantation **(C)**. **Strong Recommendation**.

#### Viral infections

Viral infections may also be transmitted through transplanted organs; however, there are many case series describing donors who were positive for several viruses, with a reduced risk especially when the recipients were also positive for the same viruses and preventive measures were implemented **(C)**.^(130)^ Consistently, organs from donors with evidence of resolved hepatitis B virus (HBV) infection (positive anti-HBc alone or associated with anti-HBs) have been transplanted into HBV carriers or immune patients (positive anti-HBs) with a minimum risk of infection transmission or reactivation with the implementation of specific antiviral prophylaxis **(C)**.^(144-146)^ Similarly, the liver and kidneys of hepatitis C virus (HCV)-positive donors were also transplanted into HCV-positive recipients with persistent viremia; the results evidenced a satisfactory risk-benefit ratio in terms of survival compared with the remaining recipients on the transplantation waiting list **(C)**.^(147,148)^ Herpesviruses (CMV, human herpes simplex virus (HSV), EBV, human herpesvirus-6 (HHV-6)) can also be transmitted via transplantation; generally, post-transplantation primary infection, i.e., in previously seronegative patients, is associated with a higher risk of clinical manifestations and severity. However, prevention might be achieved by prophylaxis or preemptive therapy, enabling transplantation between herpesvirus serodiscordant donor-recipient pairs **(B)**.^(149-151)^ The transplantation of organs from HIV-positive donors into HIV-positive recipients is currently a subject of much debate. This option has been tested in slightly more than 30 patients from South Africa with satisfactory preliminary results; however, more data are needed before this indication is considered **(C)**.^(152)^

Serology testing for HBV, HCV and HIV infection might be negative during the window period. Thus, NAT has been recommended, especially for patients at high-risk for these infections **(D)**.^(130)^

**Recommendation:** All donors must be subjected to serologic testing for the following viruses: HBV, HCV, HIV, HTLV-I/II, CMV, HSV and EBV** (A)**. **StrongRecommendation**.

**Recommendation:** Donors at high risk for infection (Table 4) with HBV, HCV or HIV can also be assessed by NAT where available** (C)**. **Weak Recommendation**.

**Recommendation:** Liver or kidneys from HCV-positive donors may be transplanted into HCV carriers** (D)**. **Strong Recommendation**.

**Recommendation:** Donors who are anti-HBs-positive alone (vaccinated) may donate all their organs to any recipient independently of the serologic status of the latter** (A)**. **Strong Recommendation**.

**Recommendation:** Donors who are anti-HBs and anti-HBc-positive or anti-HBc-positive alone (HBsAg and anti-HBs-negative) may donate organs to HBV carriers and recipients with evidence of immunity (anti-HBs-positive). These cases require post-transplant prophylaxis** (C)**. **Strong Recommendation**.

**Recommendation:** According to the judgement of the transplant team, HBsAg-positive individuals may be considered kidney donors for HBsAg-positive or anti-HBs-positive (immune) recipients with post-transplant prophylaxis** (D)**. **Weak Recommendation**.

**Recommendation:** Positive CMV, HSV or EBV serology is not a contraindication to donation. The serologic status is useful to implement post-transplant preventive measures **(B)**. **Strong Recommendation**.

**Recommendation:** Organs from HIV or HTLV-I/II-positive donors should not be used** (C)**. **Recommendation Strong**.

#### Fungal infections

The presence of systemic invasive fungal infections in potential donors is generally a contraindication to donation. Endemic mycoses, such as histoplasmosis and paracoccidioidomycosis, are more difficult to diagnose, and cases of transmission have been reported. There are no standard recommendations for the assessment and selection of this type of donor **(C)**.^(131,153)^

**Recommendation:** Organs from donors with invasive fungal infections should not be used** (C)**. **Strong Recommendation**.

**Recommendation:** The investigation of clinical and epidemiological data relative to endemic mycoses should be performed for all donors **(D)**. **Weak Recommendation**.

#### Parasitic infections

The risk of transmission of toxoplasmosis is a highly relevant issue, especially in heart transplantation. This risk is significantly increased when the donor is seropositive and the recipient is seronegative. While donation is not contraindicated in this case, the recipient must receive prophylaxis **(B)**.^(154)^ Chagas disease is another parasitic infection that has received much interest. Inadvertent transmission has been reported; all donors from endemic areas, such as Brazil, must be subjected to serologic testing. In small case series involving kidney transplantation from seropositive donors to seronegative recipients and post-transplant prophylaxis with benznidazole, no evidence of transmission was detected **(C)**.^(155,156)^ However, this option cannot be universally recommended; when adopted in urgent cases, the recipient must be periodically assessed for acute infections using tests that are able to detect high levels of parasitemia even during prophylaxis **(D)**.^(157)^

**Recommendation:** Serologic testing for toxoplasmosis and Chagas disease must be performed for all donors **(A)**. **Strong Recommendation**.

**Recommendation:** Organs from toxoplasmosis-seropositive donors may be used. Specific prophylaxis is recommended for seronegative recipients, especially in heart transplantation **(B)**. **Strong Recommendation**.

**Suggestion:** Organs from Chagas disease-seropositive donors may be considered for both seropositive and seronegative recipients. Recipients must be subjected to prophylaxis and/or monitoring for acute disease or post-transplant reactivation **(D)**. **Weak Recommendation**.

### 21. How to deal with donors with a history of neoplasia?

**Comment:** Accidental transmission of neoplasms from donors to recipients is rare.^(73,158)^ According to a United Network for Organ Sharing (UNOS) report, 35,503 deceased donors and 109,749 transplanted organs were assessed from April 1994 through December 2000, among which 9 donors transmitted neoplasms (donor transmission rate of 0.025%) to 12 recipients (organ transmission rate of 0.01%).^(159)^ However, due to the serious consequences, all potential donors must be subjected to careful investigation to avoid the occurrence of inadvertent transmission.^(158-161)^ Donors diagnosed with neoplasms should not be considered for organ **(D)**^(73,160,162,163)^ or tissue and cell **(D)**^(164,165)^ donation, except in cases of tumors with a low degree of malignancy or localized neoplasms as follows **(D)**:^(73,160,162-165)^

- Skin tumors, such as basal cell and squamous cell carcinoma;
- Carcinomas *in situ*, such as cervical carcinoma *in situ*;
- Kidney tumors diagnosed during removal or implantation, which may be accepted when their size is ≤ 4cm, they exhibit Fuhrman grade I-II and their margins are free **(C)**.^(73,162,166,167)^
- Primary CNS tumors, according to the Council of Europe recommendations (Table 5):^(74)^
  Group 1 - Metastases outside the CNS are rare; organs may be considered for donation.
  Group 2 - There is risk of transmission when other risk factors are also present; organs may only be considered for donation in the absence of these risk factors.
  Group 3 - There is a considerable risk of transmission; organs may only be used for urgent cases and with due communication to the recipients.

Specifically in the case of cornea donation, donors with malignant neoplasms can be assessed and considered, excluding those with retinoblastoma, blood cancer or eye anterior segment tumors **(D)**.^(165)^

Although there are some reports of the transmission of primary CNS tumors to recipients,^(159,168-175)^ data for transplants performed in the 1990s recorded in registries from the United Kingdom,^(175)^ Australia, New Zealand,^(176)^ Czech Republic^(177)^ and Spain^(178)^, i.e., prior to publication of the Council of Europe recommendations, do not contain a single instance of tumor transmission. According to UNOS, in the United States, of 175 recipients and donors with glioblastoma multiforme, transmission occurred in three recipients (1.7%), all from a single donor.^(179)^ Based on these data, the Advisory Committee on the Safety of Blood, Tissues and Organs concluded that the risk of dying while still on the waiting list is higher than the risk of the transmission of primary CNS tumors. Consequently, since 2012, these tumors were no longer considered a contraindication for donation independently of their histological type, and the donors are considered "marginal".^(180)^ This recommendation is currently applied in the United Kingdom only, and potential recipients are informed as to the small (but definite) risk of transmission, as well as to their survival odds if they decided to remain on the waiting list.^(181)^

The main risk factors for the transmission of primary CNS tumors are^(162,168,176)^ histological type and malignancy grade; previous history of craniotomy or stereotactic surgery; ventricular systemic shunt; previous history of chemotherapy or radiotherapy; disease duration; and length of survival after surgery.

Regarding donors who had cancer in the past, the current evidence does not suffice to recommend any definite tumor-free period as acceptable for organ donation. In addition, independently from the time since the onset of disease, neoplasms such as breast cancer, soft tissue sarcoma and melanoma develop late metastases more frequently, with a consequent higher risk of tumor transmission.

Donors must always be carefully assessed (clinical history, physical examination, auxiliary tests and surgical inventory during organ removal) to avoid the accidental transmission of neoplasms (see question 15):^(73,158,182)^

- Tumor markers should be considered in very specific situations only:
  a) Beta-human chorionic gonadotropin (beta-hCG) in women of reproductive age because it is elevated in choriocarcinoma **(D)**.^(73)^
  b) Prostate-specific antigen (PSA) has no indications **(D)**.^(183)^
  c) Other markers should be considered when there is consistent suspicion of a tumor or to establish the progression or possible relapse of a previous neoplasm **(D)**.^(73)^
- Histopathological examination is indicated in the following three situations **(D)**:^(73)^
  a) Tumor or suspicious lymph node found during organ removal surgery (frozen section analysis).
  b) Brain death caused by an intracranial lesion suspicious for metastasis, or to define the malignancy grade of the primary tumor (sections are frozen for 2-3 hours and then embedded in paraffin for 24 hours).
  c) When prostate cancer is suspected, the organ is removed en bloc for frozen section analysis, followed by a complete histopathological examination.

**Recommendation:** Each instance must be assessed on a case-by-case basis, weighing the donor tumor transmission risk versus the patient's urgency and risk of dying on the waiting list **(D)**. **Strong Recommendation**.

**Recommendation:** The donation of organs, tissues and cells is contraindicated for donors with a past history of breast cancer, melanoma, soft tissue sarcoma and blood cancers (independent from the time elapsed since the onset of disease). A possible exception might be patients with an extremely urgent need for transplantation **(D)**. **Strong Recommendation**.

**Recommendation:** Donors with a history of other types of neoplasms with a disease-free period of 3, 5 or even 10 years without tumor relapse might be accepted. The decision is made on a case-by-case basis according to the tumor type and characteristics **(D)**. **Strong Recommendation**.

**Recommendation:** The decision to accept donors with primary CNS tumors must be based on a judicious analysis following the classification formulated in the Council of Europe recommendations (Table 5) **(D)**. **Strong Recommendation**.

**Recommendation:** Beta-hCG must be measured in women of reproductive age. All other tumor markers should only be analyzed when the clinical data are suspicious for tumors **(D)**. **Weak Recommendation**.

### 22. How are patients at high risk for the transmission of viral diseases characterized? What precautions are needed in the assessment of high-risk patients?

**Comment:** High-risk donors are those who carry an increased risk of HIV, hepatitis B and C transmission during the window period, i.e., within the interval from acquisition to serologic detection of infection, and are included within the risk categories formulated by the Organ Procurement and Transplant Network (OPTN). The criteria defining high-risk donors are described in table 4 **(B)**.^(113)^ The risk of organs from these donors to transmit infection is higher compared with other donors. While this risk is actually low, it is not insignificant.

In a systematic review of donors at high risk for HIV transmission, risks ranged from 0.09 to 12.1 per 10,000 donors based on enzyme-linked immunosorbent assay (ELISA) and from 0.04 to 4.9 per 10,000 donors based on NAT. Injection drug users have the greatest risk of window period infection compared with the other high-risk donor categories **(B)**.^(114)^ Another systematic review also concluded that the risk of hepatitis C during the window period was significant among injection drug users **(B)**.^(113)^ Nevertheless, for many recipients, the risk of infection during this time did not approach the risk of dying while on the waiting list **(D)**.^(184)^

The risk of window period transmission by high-risk donors should be assessed relative to the recipients' risk of dying while on the waiting list **(C**).^(185)^ The final decision should be made by the transplantation team and the recipient upon informed consent **(D)**.^(186)^

In general, the performance of NAT for HIV, HBV and HCV is recommended in the case of high-risk donors to reduce the risk of transmission and increase the number of transplantable organs **(D)**.^(112,186)^

**Recommendation:** Potential donors at high risk of transmission of viral infections should be classified according to the categories formulated by OPTN (Table 4) and assessed in terms of the increased risk of recent infection with HIV, HBV and HCV **(D)**. **Strong Recommendation**.

**Recommendation:** The risk/benefit ratio should be carefully assessed by the transplantation team in the case of high-risk donors. The use of organs from high-risk patients requires informed consent by the recipients **(D)**. **Weak Recommendation**.

**Recommendation:** Care providers at critical care services must not rule out any potential donor; this decision is an exclusive attribution of transplant center coordinators and transplantation teams **(D)**. **Strong Recommendation**.

## PART 4: ORGAN-SPECIFIC CONTRAINDICATIONS

### 23. What conditions represent organ-specific contraindications to kidney donation? How are ideal and marginal kidneys characterized?

**Comment:** The donation of kidneys for transplantation may be contraindicated based on the (1) risk of disease transmission, (2) donor's kidney function, (3) donor's age and (4) histological condition of the kidneys.

1. The main absolute contraindication to donation is a high risk of transmission of infectious or neoplastic diseases with a poorer prognosis or progression than maintenance of renal replacement therapy - see question 19 **(B)**.^(187)^

2. The donor's vascular state and kidney function:

a) Initial (upon admission or at event onset) and final (last measurement before organ removal) creatinine (Cr) must be assessed.
b) The initial Cr 1.5mg/dL **(D)**^(188)^ or 2mg/dL **(D)**^(189)^ is considered the upper limit to define kidneys as adequate for donation **(D)**.^(190)^ Higher values characterize expanded-criteria kidneys or preclude their use.
c) Some researchers recommend the estimated glomerular filtration rate calculated using the Cockroft-Gault formula as a parameter, in which Cr is adjusted for weight, gender and age. However, this parameter has limited use in the case of critically ill patients.^(191)^ For patients with small variations in plasma Cr relative to baseline, the estimated Cr clearance must be ≥50 mL/min **(D)**.^(94)^

However, there are reports of satisfactory outcomes of kidney transplants from donors with ongoing acute kidney failure **(C)**.^(71,91,95,192-194)^

3. Donor's age: There is no current absolute contraindication to kidney transplantation as a function of the donor's age. However, kidneys from donors greater than 70 years old should be considered expanded-criteria kidneys, and transplantation is associated with a higher risk of death and graft loss, especially when the recipients are less than 60 years old **(B)**.^(95)^

4. Histology: When available, glomerular sclerosis > 20% using a sample from both kidneys containing more than 25 glomeruli **(D)**^(94)^ might represent a contraindication to donation. Possible indications for intraoperative kidney biopsy at the time of procurement are as follows **(D)**:^(190)^

i) Donor age over 65 years old.
ii) Cr over 1.5mg/dL.
iii) History of systemic arterial hypertension.
iv) Presence of diabetes*.*
v) Urinalysis with abnormalities suggestive of glomerular disease.

The following might be considered marginal or expanded-criteria donors: **(D)**.^(94,187)^

a) Age over 70 years old and no other risk factors.
b) Age of 60 to 70 years old, with a history of diabetes, systemic arterial hypertension, significant proteinuria (over 1g/24 hours) or signs of hypertension- or diabetes-induced target-organ injury.
c) Consider double kidney transplantation or discarding the organ when the glomerular filtration rate is less than 50mL/min.
d) Kidney biopsy showing glomerular sclerosis ranging from 5% to 20% in a sample from both kidneys containing more than 25 glomeruli.

**Recommendation**: Kidneys are contraindicated for donation whenever eventual transmission of infectious or neoplastic disease is associated with poorer prognosis or progression compared to the existing kidney disease **(D)**. **Strong Recommendation**.

**Recommendation:** An initial Cr exceeding 2.0mg/dL is an *a priori* contraindication to donation; alternatively, the kidneys may be subjected to macroscopic and microscopic assessment **(D)**. **Strong Recommendation**. Kidneys from donors with acute kidney failure, as demonstrated by elevation of the final Cr, may be accepted for donation **(C)**. **Strong Recommendation**. In some cases, systematic kidney biopsy might contribute to the determination of absolute contraindications **(D)**. **Strong Recommendation**.

**Recommendation:** The characteristics of expanded criteria kidney donors are age > 70 years old (without any other risk factors) or 60 to 70 years old with diabetes; systemic arterial hypertension or proteinuria > 1g/24 hours; Cr > 2mg/dL; glomerular filtration rate < 50mL/min; and glomerular sclerosis ranging from 5% to 20% in the kidney biopsy (sample from both kidneys containing more than 25 glomeruli) **(D)**. **Weak Recommendation**.

**Recommendation:** The characteristics of ideal kidney donors are age < 60 years old and baseline Cr < 1.5 to 2mg/dL **(D)**. **Weak Recommendation**.

### 24. What conditions represent contraindications to liver donation? How are ideal and marginal liver donors characterized?

**Comment:** The main absolute contraindication to liver donation is a high risk of transmission of infectious or neoplastic diseases with a poorer prognosis or progression compared with the existing liver disease - see question 19 **(B)**.^(195)^ Special attention should be paid to possible liver metastases of choriocarcinoma, which are macroscopically similar to liver hemangioma (a benign tumor that is highly prevalent in young women). At times, brain metastases of choriocarcinoma cause CNS bleeding and brain death, resulting in the transplantation of livers with metastases of choriocarcinoma, as described in the literature.

Cirrhosis of the liver is an absolute contraindication for donation, and it is ill-advised to transplant a fibrosis grade 3 or higher liver. Livers with necrosis > 20% on biopsy, such as in the case of cocaine overdose, are unacceptable for transplantation **(C)**.^(196)^ Macrovesicular steatosis > 60% together with a cold ischemia time longer than 12 hours is associated with a high risk of poor graft survival **(B)**.^(197)^ Severe steatosis combined with an age > 65 years old and the presence of some risk factor and/or gamma-GT three times above the normal value and/or prothrombin time < 40% and/or platelet count < 60.000/mm^3^ is also a contraindication to donation independent of the cold ischemia time **(C)**.^(100)^ Although the age of the donor has little weight by itself in the decision to use a liver, livers from donors greater than 90 years old tend to be discarded. The presence of infection, lesions or severe trauma represent absolute contraindications. Elevated transaminases indicate hepatocellular injury; levels exceeding 300U/L and tending to increase should be analyzed on a case-by-case basis **(D)**.^(100,198,199)^

While some clinical and laboratory data might be little relevant when assessed separately, combinations of variables may result in situations that are difficult to assess in clinical studies.

Steatosis, length of hospital stay, age, ischemia (due to high-dose vasopressors), AST/ALT, gamma-DT and cold ischemia time must be analyzed jointly. Abnormalities in any of these factors alone rarely represents an absolute contraindication to donation. Liver trauma is a frequent occurrence among deceased donors in whom TBI was the cause of brain death. Only very severe liver injuries represent a contraindication to donation, such as lesions with active bleeding, segmental pedicle injury and extensive parenchymal avulsion.

One should always weight the risk of liver graft nonfunction against the risk of the death of the recipient while on the waiting list. Liver graft nonfunction requires retransplantation, or the recipient will die. Although the current trend is to increase the flexibility of the criteria to accept non-ideal grafts, the incidence of primary nonfunction has actually decreased. Additionally, the mortality of patients on the waiting list is continuously increased and is estimated to be at least eight times higher compared with primary liver nonfunction. For validation, poor liver function in the immediate postoperative period increases the length of stay at the ICU and the hospital, with a consequent increase in the cost of liver transplantation.

**Recommendation:** Liver donation is contraindicated whenever eventual transmission of an infectious or neoplastic disease is associated with poorer prognosis or progression compared with the existing liver disease **(B)**. **Strong Recommendation**.

**Recommendation:** Organ-specific contraindications to liver donation for transplantation are cirrhosis, infections, parenchymal or pedicle lesion or trauma. The transaminase levels themselves do not determine contraindication. Values exceeding 300U/L and tending to increase should be evaluated by the transplantation team **(D)**. **Strong Recommendation**.

**Recommendation:** Age alone does not determine contraindication to liver donation **(D)**. **Strong Recommendation**.

**Recommendation:** Biopsy of the potential donor's liver combined with other variables, such as cold ischemia time, age and laboratory tests, may guide the donation decision **(D)**. **Strong Recommendation**.

**Recommendation:** The characteristics of expanded-criteria liver donors are age > 60 years old, cold ischemia time > 12 hours, steatosis > 30% and length of stay at the ICU > 5 days. The expanded criteria may in no case overrule the absolute contraindications to liver donation **(D)**. **Weak Recommendation**.

**Recommendation:** The characteristics of ideal liver donors are an age up to 50 years old, length of stay at the ICU <5 days, no relevant abnormalities on liver tests and imaging until removal and/or infusion of vasopressors at a low dose **(D)**. **Weak Recommendation**.

### 25. What conditions represent contraindications to heart donation? How are ideal and marginal heart donors characterized?

**Comment:** Successful heart transplants are initiated by adequate donor selection. Among the many variables involved in the various steps of the transplantation process, we argue that the point of departure from success is the exchange of information that begins with the very first telephone call, at which moment the agency responsible for organ procurement and the surgeon who will perform the transplant must establish a close collaboration. The two central and unifying concepts in the selection of a donor heart are (1) the quality of the heart and (2) matching of the donor heart to the individual needs of the recipient **(D)**.^(200)^ The use of marginal donors is only justified when the risk of patient death from heart disease is higher than that due to donor-related reasons **(D)**.^(101)^ However, the current scenario is characterized by an increasing need to use organs from marginal donors, possibly as a result of a shift in the profile of patients on the waiting list. The prevalence of patients bridged with univentricular or biventricular assist devices is increasing worldwide, as well as recipients with pulmonary hypertension and kidney dysfunction. Long waiting times and the critical state of many patients on the waiting list are some of the factors that impose considerable pressure on medical staff to opt for marginal donors. For these reasons, a critical and careful analysis of the characteristics defining ideal and marginal donors and of the contraindications to donation by transplantations teams has paramount importance. Such an analysis should lead transplantation teams to the best decision regarding whether to accept a given heart based on the optimal survival expectancy, i.e., to use an organ from a marginal donor or to keep the recipient on the waiting list until an ideal donor appears.

The main absolute contraindication to heart donation is the high risk of transmission of infectious or neoplastic diseases with a poorer prognosis or progression compared with the existing heart disease - see question 19.

Despite the higher mortality of recipients of hearts from donors over 64 years of age, age does not represent an absolute contraindication to heart donation for transplantation **(D)**.^(101)^

Elevation of cardiac enzymes is extremely common among potential heart donors. Serum troponin levels alone should not be used as a contraindication to transplantation **(D)**.^(201)^

Ventricular hypertrophy alone is not associated with a higher post-transplantation risk of mortality** (B)**^(202)^ but should be considered when associated with other risk factors, such as a donor age over 55 years old and an ischemia time exceeding 4 hours **(C)**.^(202)^

Parameters that are considered unfavorable for heart transplantation are hemorrhagic stroke as a cause of donor death, age > 50 years old, prolonged ischemia time (more than 240 minutes) **(D)**,^(101)^ vasopressor dose > 15mcg/kg/minute and donor weight/recipient weight ratio < 0.8.

There is a consensus in the literature regarding the relationship between an older donor age and higher post-transplant mortality. We observed variations in the reported age limits. The review published by Kilic et al. states that, traditionally, accepted donors are <55 years old **(D)**.^(200)^ After collecting and analyzing data from transplant centers in the state of São Paulo, Brazil, Fiorelli et al. concluded that a donor age > 40 years is a relevant risk factor for post-transplant survival; however, caution is needed regarding the donor populations analyzed in that study (**C**).^(203)^ A single-center study conducted in Portugal (**C**)^(204)^ found that the incidence of acute rejection and 5-year mortality was similar between the group of recipients of hearts from donors aged 50 years or older (mean of 52 years old) and those who received hearts from donors under 40 years of age (mean of 28 years old). Of relevant interest, the ischemia time was significantly shorter among the older donors, i.e., the total ischemia time was < 60 minutes for 58% of the older donors. The authors of a multicenter study conducted in Spain reached similar conclusion because they did not find any difference in the rate of acute rejection and global mortality between recipients of hearts from donors over or under 50 years of age after adjustment for possible confounding factors, such as the age and immunosuppressive regime of the recipient **(C)**.^(205)^

While bacteremia is not a contraindication for transplantation, patients with septic shock are not acceptable as donors **(D)**.^(132,206,207)^ A recent study assessing 26,813 heart transplants in the United States, including 995 cases in which hearts from donors with positive blood cultures were used, showed that this variable did not influence the survival of recipients, although that population of recipients exhibited greater morbidity at the time of transplantation **(D)**.^(208)^ The use of hearts from potential donors infected with *Trypanosomacruzi* **(C)**^(206,209)^ or chronic hepatitis B and C **(D)** is contraindicated.^(206)^ The following infections represent absolute contraindications to donation: HIV, HTLV-1, systemic viral infection (e.g., rubella, rabies, adenoviruses, enteroviruses, parvoviruses) and viral meningoencephalitis **(D)**.^(206)^ There are no recommendations in the literature related to donation by individuals with a recent infection with dengue or influenza. CMV and syphilis are not contraindications to heart transplantation **(D)**.^(206)^

Vasopressors (dopamine, noradrenaline and vasopressin) are commonly administered to potential donors and are not a contraindication of heart donation for transplantation by themselves. Only patients with refractory shock, i.e., who maintain severe arterial hypotension despite fluid resuscitation, high-dose vasopressors and hormone replacement therapy and particularly those with multiple organ dysfunction, are not accepted as heart donors **(D)**.^(132,206,207)^ Structural abnormalities and irreversible contractility disorders should be considered contraindications **(C)**.^(201-203,208-215)^

**Recommendation:** Heart donation is contraindicated whenever the eventual transmission of an infectious or neoplastic disease is associated with a poorer prognosis or progression compared with the existing heart disease **(B)**. **Strong Recommendation**.

**Recommendation:** Organ-specific contraindications to heart donation for transplantation are as follows: infection with *T. cruzi*, severe heart defects, left ventricular hypertrophy > 13mm, coronary artery disease affecting more than one vessel, malignant arrhythmias and persistent circulatory instability despite maximum hemodynamic therapy **(D)**. **Strong Recommendation**.

**Recommendation:** A high-dose vasopressor or beta-agonist therapy may compromise the success of heart transplantation, but it does not represent a contraindication **(D)**. **Weak Recommendation**.

**Recommendation:** Elevated biomarkers are not an absolute contraindication to heart transplantation; their relationship with persistent myocardial dysfunction must be analyzed **(D)**. **Strong Recommendation**.

**Recommendation:** The characteristics of expanded-criteria heart donors are as follows: hemorrhagic stroke as the donor cause of death, age > 50 years old, cold ischemia time > 240 minutes, use of high-dose vasopressors and donor weight/recipient weight ratio < 0.8 **(D)**. **Weak Recommendation**.

**Recommendation:** The characteristics of ideal heart donors are as follows: left ventricular ejection fraction > 50%, absence of structural and contractility abnormalities, cardiac index > 2.5L/min/m^2^ and pulmonary wedge pressure (PAWP) ≤ 15mmHg. Donation should not be ruled out even when these values are not attained **(D)**. **Weak Recommendation**.

### 26. What conditions represent contraindications to lung donation? How are ideal and marginal lung donors characterized?

**Comment:** The criteria that are currently used for the acceptance of lung donors are based on clinical impressions instead of consolidated evidence. Even when chest radiographs suggest bilateral lung lesions or donors have some infection, the final decision to accept the donation is made by the transplantation team. The final decision regarding the use of lungs for transplantation should be made on a case-by-case basis based on the characteristics of the donor and recipient **(D)**.^(102,112,113,216-219)^ Experience gathered over the previous 20 years has shown that many lungs that were dismissed through the application of ideal criteria could have been used without any harm to the recipients. Expansion of the traditional criteria for donation is clearly necessary to reduce the shortage of lungs for transplantation. However, follow-up studies are needed to validate the efficacy and safety of the expanded criteria. Within this context, the criteria for lung donation have been expanded in recent years.

The currently accepted **expanded criteria** for lung donors are as follows: age > 55 years old, chest radiograph with localized or unilateral abnormalities, smoking < 40 packs/year, PaO_2_/FiO_2_ > 250 (with positive end-expiratory pressure - PEEP = 5cmH_2_O), absence of extensive thoracic trauma, no history of cardiothoracic surgery, a compatible blood type, mechanical ventilation < 5 days and the presence of upper airway secretions **(B)**.^(216,217)^

**Ideal donors** should have an age of < 55 years, compatible blood type, normal chest radiograph, smoking < 20 packs/year, PaO_2_/FiO_2_ > 300 (with PEEP = 5cmH_2_O), an absence of significant thoracic trauma, no history of cardiothoracic surgery, negative airway microbiological findings, normal bronchoscopy results, no signs of bronchial aspiration or infection, mechanical ventilation < 3 days, no history of active or recent neoplasm (except for skin squamous cell or basal cell carcinoma, localized cervical cancer, primary brain neoplasms with low metastatic potential and in the absence of invasive procedures involving the skull or brain), no infection with HIV, HBV or HCV and no uncontrolled sepsis **(B)**.^(102,113,216)^

The main **absolute contraindication** to lung donation is the high risk of transmission of infectious or neoplastic diseases with a poorer prognosis or progression compared with the existing lung disease - see question 19 **(D)**.^(76,107,108,114-118,175,220-224)^

Additionally, the presence of bilateral lung abnormalities on chest radiographs **(C)**^(219,220)^ is a contraindication to lung transplantation. According to some reports, grafts from donors with asthmatic lungs might be associated with poor short- and long-term functional outcomes **(D)**.^(219)^

The development of *ex vivo* perfusion systems for the reconditioning of injured lungs before implantation provides a new tool for the use of marginal donors or lungs that were initially considered to be inadequate for transplantation **(A)**.^(225,226)^ While currently available at a small number of centers*, ex vivo* lung perfusion significantly improves oxygenation and enables the successful transplantation of lungs that were initially considered to be inadequate **(A)**.^(225,227,228)^

**Recommendation:** Lung donation is contraindicated whenever the eventual transmission of an infectious or neoplastic disease is associated with a poorer prognosis or progression compared with the existing lung disease **(B)**. **Recommendation Strong**.

**Recommendation:** The following are organ-specific contraindications to lung donation: bilateral lung abnormalities on chest radiographs and a history of bronchial asthma **(C)**. **Strong Recommendation**.

**Recommendation:Expanded criteria** lung donors exhibit an age > 55 years old, localized or unilateral abnormalities on chest radiograph, smoking < 40 packs/year, PaO_2_/FiO_2_ > 250 (with PEEP = 5cmH_2_O), absence of extensive thoracic trauma, compatible but not identical blood type, mechanical ventilation < 5 days and the presence of airway secretions **(B)**. **Weak Recommendation**.

**Recommendation:** Ideal lung donors exhibit an age < 55 years old, a compatible blood type, normal chest radiograph, smoking < 20 packs/year, PaO_2_/FiO_2_ > 300 (with PEEP = 5cmH_2_O), absence of extensive thoracic trauma, no history of cardiothoracic surgery, negative airway microbiologic findings, normal bronchoscopy results, no signs of bronchial aspiration or infection and mechanical ventilation < 3 days** (B)**. **Weak Recommendation**.

### 27. What conditions represent contraindications to pancreas donation? How are ideal and marginal pancreas donors characterized?

**Comment:** Because transplantation of the pancreas is not considered essential to save the life of the recipient, an adequate clinical and laboratory assessment of potential donors is crucial to obtain high-quality grafts and to avoid the transmission of infectious or neoplastic diseases to recipients.

Due to the shortage of organs, the criteria for acceptance have been increasingly expanded, and "marginal" donors are currently accepted. There are few absolute contraindications to pancreas donation. The main absolute contraindication is the risk of transmission of infectious or neoplastic diseases with a poorer prognosis or progression compared with the existing disease - see question 19. Other situations also represent contraindications to donation, all of which are related to the global assessment performed by the organ procurement team or established by surgeons at the time of organ removal **(D)**.^(69,229)^ Among concerns regarding the specific case of pancreas donation, the following conditions are dominant: confirmed diabetes, pancreatitis (acute/chronic), considerable pancreatic steatosis or edema and previous pancreatic surgery.

Despite the few criteria for the absolute contraindication of pancreas donation notwithstanding, up to 43% of the offered organs are refused and 23% discarded after macroscopic assessment, supporting the subjectivity of the contraindication **(B)**.^(230)^ The demographic variables that define an almost ideal pancreas donor are as follows **(B)**:^(101)^ donation after brain death, age range from 10 to 40 years old, body mass index (BMI) < 27.5/m^2^ and brain death unrelated to cerebrovascular causes.

It should be noted that the Brazilian Ministry of Health ordinance no. 2,600, from October 2009 establishes an age of 50 years and a BMI of 30kg/m^2^ as upper limits for pancreas donation **(B)**.^(76)^

Recent data published by the International Pancreas Transplant Registry (IPTR) confirmed the relevance of demographic variables related to donors, especially younger age and brain death not due to cerebrovascular causes in relation to increased long-term (> 1 year) graft survival (**B**).^(231)^

Due to the high rate of graft loss within 1 year, the acceptance of organs depends on the personal interpretation made by the surgeon in charge of the transplantation surgery, resulting in a high proportion of refusals, which may not always be grounded on formal criteria. Some scores were formulated in an attempt to systematize the acceptance or refusal of donated pancreases, such as the Preprocurement-Pancreas-Suitability-Score (P-PASS) developed by the Eurotransplant Pancreas Advisory Committee. This score is based on donor data obtained at the time of notification; the total score ranges from 9 to 28, and pancreases with a P-PASS over 17 are three times more likely to be refused **(B)**.^(232)^ The parameters considered are age, BMI, length of ICU stay, cardiac arrest, sodium, amylase, lipase and catecholamine levels. While the P-PASS was developed to estimate the odds of pancreas acceptance, some centers sought to establish whether it might also predict graft survival, with conflicting results. A Eurotransplant retrospective assessment found that the 1-year pancreas graft survival in patients subjected to simultaneous pancreas-kidney transplantation was significantly higher among those who had received organs from donors with a P-PASS < 17 **(B)**.^(233)^ However, the results of single-center studies did not show a difference in the occurrence of ischemia/reperfusion injury or 1-year graft survival as a function of the P-PASS score **(B)**.^(234-236)^

Another index was developed based on a retrospective analysis of the Scientific Registry of Transplant Recipient data together with the Pancreas Donor Risk Index (P-DRI), which consists of an assessment of the organ quality to predict the 1-year graft survival **(B)**.^(237)^ The P-DRI considers gender, age, height, race, BMI, cause of brain death, Cr, preservation time and donation after cardiac death. An ideal donor has a P-DRI = 1. An age > 45 years old, BMI > 30kg/m^2^ and donation after cardiac death increase the P-DRI to 1.56, 1.17 and 1.39, respectively, which are scores that are associated with greater 1-year graft loss rates. However, the results of single-center studies have provided conflicting results relative to some of the considered parameters because the outcomes were similar for patients with a BMI above and below 30kg/m^2^ **(B)**^(238)^ and for donors older and younger than 45 years old **(B)**.^(239,240)^ Therefore, many decisions remain necessary regarding concerns related to criteria for the indication and contraindication of pancreas donation. A major trial, the EXPAND study (Extended Pancreas Donor Program), which is currently underway, was designed to assess extended criteria for the donation and acceptance of pancreases and to compare the related morbidity and mortality to those associated with presently used criteria **(A)**.^(241)^

**Recommendation**: Pancreas donation is contraindicated whenever the eventual transmission of an infectious or neoplastic disease is associated with a poorer prognosis or progression compared with the existing pancreas disease **(B)**. **Strong Recommendation**.

**Recommendation:** Organ-specific contraindications for pancreas donation include confirmed diabetes, macroscopic evidence of acute or chronic pancreatitis, considerable pancreatic steatosis or edema and previous pancreatic surgery **(D)**. **Strong Recommendation**.

**Recommendation:** The characteristics of the ideal pancreas donor are an age ranging from 10 - 40 years old, BMI < 27.5kg/m^2^ and brain death unrelated to cerebrovascular causes **(B)**. **Weak Recommendation**.

**Recommendation:** The characteristics of expanded criteria pancreas donors have not yet been established **(D)**. **Strong Recommendation**.

### 28. What conditions represent contraindications to intestine donation?

**Comment:** The criteria for donor selection are similar to those for pancreas donors, with a special emphasis on hemodynamic abnormalities and CMV serology **(C)**.^(242-245)^

The general absolute contraindications for all organ donors also apply to intestine donors, i.e., the risk of transmission of infectious or neoplastic diseases with a poorer prognosis or progression compared with the existing enteric disease - see question 19.

Some organ-specific features concern donor age, which ideally should be less than 50 years old, hemodynamic stability and adequate blood glucose during the donor maintenance period. Although some evidence points to negative effects of donor hyperglycemia and hyperamylasemia on graft function, none of these biochemical abnormalities alone are criteria for organ refusal **(C)**.^(242)^

Donors who are administered high-dose vasopressors must be refused. One of the main assessment criteria is the macroscopic examination of the intestine during removal. The presence of turbid fluid and fibrin in the peritoneal cavity, edematous bowel loops, hematomas, mesenteric fatty infiltration and signs of poor perfusion are risk factors for post-transplant complications **(C)**.^(241-247)^

The criteria for selection of intestine donors are an age ranging from 10 to 50 years (for adult recipients), weight from 5 to 90kg (for child and adult recipients) and no history of chronic alcoholism.

**Recommendation:** Intestine donation is contraindicated whenever eventual transmission of an infectious or neoplastic disease is associated with poorer prognosis or progression compared with the existing intestinal disease **(B)**. **Strong Recommendation**.

**Recommendation:** Donation is contraindicated whenever there is macroscopic evidence of intestinal ischemia, the donor has a history of chronic alcoholism, age <10 and >50 years old (for adult receptors) and weight < 5 and > 90 kg **(C)**. **Strong Recommendation**.

### 29. What conditions represent absolute contraindications to tissue donation?

**Comment:** Tissue donation is based on the identification of potential cornea, musculoskeletal tissue (bone, tendon, meniscus and osteochondral tissue), heart valve, blood vessel and skin donors. More than 2 million tissue transplants are performed every year worldwide **(C)**.^(247)^ The process of donor identification is complex because each tissue has specific contraindications, which makes this an extremely broad-scoped subject. This situation is a consequence of the peculiarities inherent to this type of donation because, in contrast to the different organs, tissues are usually transplanted for rehabilitation purposes. As a result, the assessment and exclusion of potential donors demands the evaluation of a rather long list of contraindications and meeting quite rigorous selection standards, which are generally more extensive than those for organ donation **(C)**.^(248)^

For didactic purposes, only evidence supporting the most common absolute contraindications for donation of the aforementioned tissues are listed and discussed here because they correspond to situations in which possible donors must be mandatorily excluded from the pool of donated tissues.

Didactically, the criteria for absolute exclusion of potential tissue donors might be divided as follows: criteria related to risk factors in the donor, including information collected from medical records, families and physical examination of deceased donors and laboratory criteria, with an emphasis on serologic testing.

The risk factors that are subsequently described are considered absolute contraindications for tissue donation due to an increased risk of disease transmission:

1. Factors associated with high-risk behaviors in the previous 12 months (these criteria apply to all tissues):

- **Injection** drug use, due to an increased risk of HIV, hepatitis B and C infection through sharing of putatively contaminated needles** (C)**.^(249)^ The high risk of transmission of viral infections among blood donors who are drug users has been well established in epidemiological surveys since the 1990s **(C)**,^(250)^ and for safety reasons, the evidence has been extrapolated to tissue donors despite an absence of scientific evidence in this regard.
- Exchange of sex for money, sex with multiple partners or exposure to confirmed infected partners, given the higher odds of engaging in multiple unprotected sex acts. Epidemiological studies targeting non-intravenous drug-using patients attending clinics for sexually transmitted diseases (STD), such as the one by Thomas et al. **(C)**,^(251)^ demonstrated almost 20 years ago a high prevalence of HIV, HBV and HCV among individuals with risky sexual behaviors. Other studies from that time, specifically those involving sex workers, also demonstrated a high incidence of viral contamination **(C)**^(251-253)^ **(D)**.^(254)^
- Homosexual sex among men because the literature indicates that the incidence of multiple sex partners and unprotected sex is higher among males. In a cohort study conducted in 1998 investigating HIV-negative men who had sex with men, Tabet et al. found an increased incidence of STDs** (C)**.^(255)^ Another epidemiological study published the same year by Katz et al. reported similar results** (C)**.^(256)^

2. Factors associated with diagnosed or suspected diseases before donation:

- Known potentially metastatic malignancy, HIV, STD, hepatitis B or C, active tuberculosis, local or systemic fungal disease, due to the established risk of receptor contamination **(C)**. Cases with local bacterial infection are also contraindicated. Systemic bacterial infection contraindicates tissue donation in cases where sepsis was not clinically controlled (e.g.: persistent fever or temperature curve worsening, growing WBC count, progressive higher vasopressor doses) until the time of donation **(B)**.^(249,257-259)^ Chandrasekar et al. analyzed records corresponding to 1,000 tissue donors and found that 6% of them were refused because of considerable or systemic infection in 53% of cases** (C)**.^(260)^ In a study by Erbs et al. **(C)**,^(261)^ sepsis resulted in the exclusion of 29% of potential cornea donors. It is a consensus among specialists that the risks of transplantation of tissues from septic donors do not compensate for the benefits, especially given the current hypothesis that septic patients are functionally immunosuppressed **(B)**,^(262)^ increasing the risk of severe infection in the tissues of interest.

**Recommendation:** Tissue donation is contraindicated whenever eventual transmission of an infectious or neoplastic disease is associated with a poorer prognosis or progression compared with the existing disease **(B)**. **Strong Recommendation**.
